# Impact of DNA ligase 1 and IIIα interactions with APE1 and polβ on the efficiency of base excision repair pathway at the downstream steps

**DOI:** 10.1016/j.jbc.2024.107355

**Published:** 2024-05-07

**Authors:** Danah Almohdar, David Murcia, Qun Tang, Abigail Ortiz, Ernesto Martinez, Tanay Parwal, Pradnya Kamble, Melike Çağlayan

**Affiliations:** Department of Biochemistry and Molecular Biology, University of Florida, Gainesville, Florida, USA

**Keywords:** base excision repair, AP-endonuclease 1, DNA polymerase β, DNA ligase 1, DNA ligase IIIα, DNA repair, genome stability, protein–protein interaction, protein-DNA binding

## Abstract

Base excision repair (BER) requires a tight coordination between the repair enzymes through protein–protein interactions and involves gap filling by DNA polymerase (pol) β and subsequent nick sealing by DNA ligase (LIG) 1 or LIGIIIα at the downstream steps. Apurinic/apyrimidinic-endonuclease 1 (APE1), by its exonuclease activity, proofreads 3′ mismatches incorporated by polβ during BER. We previously reported that the interruptions in the functional interplay between polβ and the BER ligases result in faulty repair events. Yet, how the protein interactions of LIG1 and LIGIIIα could affect the repair pathway coordination during nick sealing at the final steps remains unknown. Here, we demonstrate that LIGIIIα interacts more tightly with polβ and APE1 than LIG1, and the N-terminal noncatalytic region of LIG1 as well as the catalytic core and BRCT domain of LIGIIIα mediate interactions with both proteins. Our results demonstrated less efficient nick sealing of polβ nucleotide insertion products in the absence of LIGIIIα zinc-finger domain and LIG1 N-terminal region. Furthermore, we showed a coordination between APE1 and LIG1/LIGIIIα during the removal of 3′ mismatches from the nick repair intermediate on which both BER ligases can seal noncanonical ends or gap repair intermediate leading to products of single deletion mutagenesis. Overall results demonstrate the importance of functional coordination from gap filling by polβ coupled to nick sealing by LIG1/LIGIIIα in the presence of proofreading by APE1, which is mainly governed by protein-protein interactions and protein-DNA intermediate communications, to maintain repair efficiency at the downstream steps of the BER pathway.

Genome instability—whether in the form of single point mutations, small insertions or deletions, or gross chromosomal rearrangements—is known to contribute to the development of disease and aging (1). Base excision repair (BER) is the critical repair mechanism for preventing the mutagenic and lethal consequences of DNA damage that arises from endogenous reactive chemical species and environmental agents (2). The BER pathway requires the coordinated action of the four core repair enzymes (3): DNA glycosylase, apurinic/apyrimidinic (AP)-endonuclease 1 (APE1), DNA polymerase (pol) β, and DNA ligase (LIG) I, or LIGIIIα. The damaged base is first removed by a lesion-specific DNA glycosylase, resulting in an AP-site in the dsDNA (4). The AP-site is then recognized by APE1, which cleaves the phosphodiester backbone at the lesion, generating a single-strand break with a 5′-deoxyribose phosphate (5′-dRP) (5). Next, polβ removes the 5′-dRP group *via* its lyase activity and subsequently catalyzes template-directed gap filling DNA synthesis through its nucleotidyl transferase activity (6). In addition to its endonuclease function, APE1 acts as a proofreading enzyme through its 3′-5′ exonuclease activity to process 3′-end of nick repair intermediate containing a damaged (*i.e.*, 8oxoG) or mismatched base incorporated by polβ (5). During the downstream steps of the BER pathway, the polβ nucleotide insertion into gap repair intermediate generates a nick product to be subsequently sealed by LIG1 or LIGIIIα that catalyze a phosphodiester bond formation between the 3′-hydroxyl (3′-OH) and 5′-phosphate (5′-P) ends to complete the repair process (7). The studies indicate that they can be interchangeable, apparently being selected, at least in part, by the choice between BER subpathways (8). The BER pathway involves a series of enzymatic steps and the network of protein–protein interactions between core BER proteins, which is critical to ensure a coordinated repair at each step (9, 10, 11). In the process referred to as “passing-the-baton,” BER proteins coordinate to hand off the DNA intermediates in a sequential manner where the product of each enzyme serves as a substrate for the next in the repair pathway (12, 13, 14). The BER pathway coordination is also facilitated by accessory non-enzymatic factor and scaffolding protein X-ray repair crosscomplementing protein 1 (XRCC1) that is critical for the recruitment of BER enzymes to the site of DNA damage (15). If this normal coordination breaks down, persistent exposure of BER intermediates may trigger harmful nuclease activities or cell death and can be converted into toxic double-strand breaks in replicating DNA, ultimately leading to genomic instability (16). While the roles of the individual enzymes in this core BER pathway are largely established, how the repair proteins function together in a multiprotein/DNA complex to facilitate the faithful channeling of DNA intermediates and repair pathway coordination is poorly understood. In the present study, we aimed to determine how the interactions of LIG1 and LIGIIIα with polβ and APE1 impact the functional coordination and processing of repair intermediates at the downstream steps of the BER pathway involving final ligation of nick repair product.

Our biochemical and structural studies reported that BER can contribute to genome instability if normal coordination breaks down at the downstream steps involving polβ and DNA ligase activities (17, 18, 19, 20, 21, 22, 23, 24, 25, 26, 27, 28, 29, 30). We demonstrated that if the 5′-dRP group is not removed by polβ, LIG1, or LIGIIIα can fail resulting in the formation of a 5′-adenylated-dRP–containing (5′-dRP-AMP) abortive repair intermediate (17, 18, 19). Similarly, the DNA ligation step of BER pathway is compromised after polβ insertion of oxidized nucleotide, 8-oxodGTP, through mutagenic base pairing with adenine, leading to ligation failure and the formation of the abortive repair intermediate with a 5′-adenylate (5′-AMP) block (20, 21). Furthermore, we have shown that noncanonical mismatches and ribonucleotides incorporated by polβ can affect the efficiency of the final steps (22, 23). Our studies also demonstrated how the disease-associated mutations linked to LIG1 deficiency syndrome and the fidelity of LIG1 could be important determinants of repair coordination (24, 25, 26). Furthermore, we reported the role of XRCC1 for facilitating the substrate-product channeling from gap filling by polβ to nick sealing by LIGIIIα at the downstream steps of the BER pathway (27, 28). Recently, our LIG1 structures demonstrated the mechanism by which the ligase active site engages with mutagenic BER intermediates, including mismatches or ribonucleotides incorporated by polβ (29, 30). However, a complete understanding of the molecular determinants that dictate BER fidelity and the impact of APE1/polβ/DNA ligase interplay on the repair pathway coordination through protein–protein interactions remain largely unknown.

The LIG1 and LIGIIIα share a highly conserved catalytic core consisting of adenylation and oligonucleotide-binding domain or OB fold as well as DNA-binding domain (DBD) and contain unrelated amino (N)- and/or carboxyl (C)-terminal regions that mediate their interactions with specific protein partners and direct them for the participation in different DNA transactions (31). For example, the amino acid residues 2 to 9, called “PCNA interacting protein box,” residing within the noncatalytic N-terminal region of LIG1 constitute the major interaction site with the interdomain connector loop of PCNA and is required for the recruitment of LIG1 to replication foci (32, 33, 34). Studies have shown that amino acid substitutions at the PCNA interacting protein box disrupt PCNA binding, abolish subnuclear targeting of LIG1 to replication site, and compromise the efficient joining of Okazaki fragments (35, 36, 37). This flexible unstructured N-terminal region also governs LIG1 interactions with other DNA replication proteins such as Rad9-Rad1-Hus1 (9-1-1), a heterotrimeric DNA sliding clamp involved in cell cycle checkpoints, replication protein A that binds to ssDNA, and replication factor C (RFC) that loads PCNA onto DNA (38). Furthermore, LIG1 interacts with polβ through this noncatalytic N-terminal domain (39, 40). LIGIIIα contains a C-terminal BRCA1 C-terminal (BRCT) domain that mediates its interaction with nonenzymatic scaffolding protein XRCC1 (41, 42). LIGIIIα also contains N-terminal extension containing a zinc-finger (ZnF) domain that has been shown to cooperate with a downstream DBD, and therefore, plays role as a “nick sensing” in nick DNA binding that increases LIGIIIα sensitivity to ssDNA breaks (43). This module, together with the catalytic core, orchestrates a dynamic switch between the initial nick sensing and the subsequent sealing in a “jack knife” fashion (44, 45).

In the present study, we measured in real time protein–protein interaction kinetics of the BER ligases using the full-length and truncated mutants of LIG1 and LIGIIIα harboring the N- and/or C-terminal regions of the proteins: LIG1 full-length (1–919), LIG1▵C-terminal (1–262) and LIG1▵N-terminal (262–919), as well as LIGIIIα full-length (1–922), LIGIIIα▵ZnF (170–922), and LIGIIIα▵BRCT (1–755). We investigated the interactions of both BER ligases with polβ and their functional interplay during the repair pathway coordination at the downstream steps involving gap filling by polβ and subsequent nick sealing by LIG1 or LIGIIIα. Furthermore, we questioned the impact of APE1 interactions with the BER ligases during processing of noncanonical nick repair intermediates containing 3′ mismatches that mimic polβ misincorporation products.

Our results demonstrated that LIGIIIα is the BER ligase that interacts more tightly with both polβ and APE1 than LIG1. We showed that these protein interactions are mediated by the N-terminal noncatalytic domain of LIG1 and the region of LIGIIIα corresponding to the catalytic core and BRCT domain. Furthermore, our results revealed relatively less amount of nick sealing products after polβ dGTP:C insertion in the presence of truncated mutants of the BER ligases, LIGIIIα lacking ZnF domain (LIGIIIα▵ZnF), and LIG1 lacking N-terminal domain (LIG1▵N-terminal), in comparison with the ligation products in the presence of the full-length proteins. Finally, we demonstrated that APE1 and LIG1/LIGIIIα could interplay on the nick repair intermediate containing noncanonical ends during the removal of 3′ mismatches *versus* ligation of nick or gap resulting in deleterious DNA intermediates. Lastly, DNA binding kinetics measurements showed that polβ and BER ligases can bind to one nucleotide gap DNA with similar efficiency, while nick DNA binding affinity of LIG1 and LIGIIIα is stronger than that of polβ. Overall, our findings could contribute to understanding how a multiprotein repair complex (APE1, polβ, LIG1, and LIGIIIα) coordinate at the downstream steps during the processing of gap and repair intermediates to maintain the BER efficiency.

## Results

### Protein interaction profile of BER ligases

We quantitatively monitored the kinetics of protein–protein interactions for LIG1, and LIGIIIα, with APE1 and polβ. Furthermore, we interrogated the interaction regions of both ligases using full-length and truncated mutants of LIG1 (▵C terminal and ▵N terminal) and LIGIIIα (▵BRCT and ▵ZnF). For this purpose, we immobilized glutathione-*S*-transferase (GST)-tag polβ or APE1 on CM5 biosensors onto which his-tag LIG1 or LIGIIIα protein was respectively passed as analytes. The interaction kinetics was measured by surface plasmon resonance (SPR) assays in real time.

**Figure 1:**
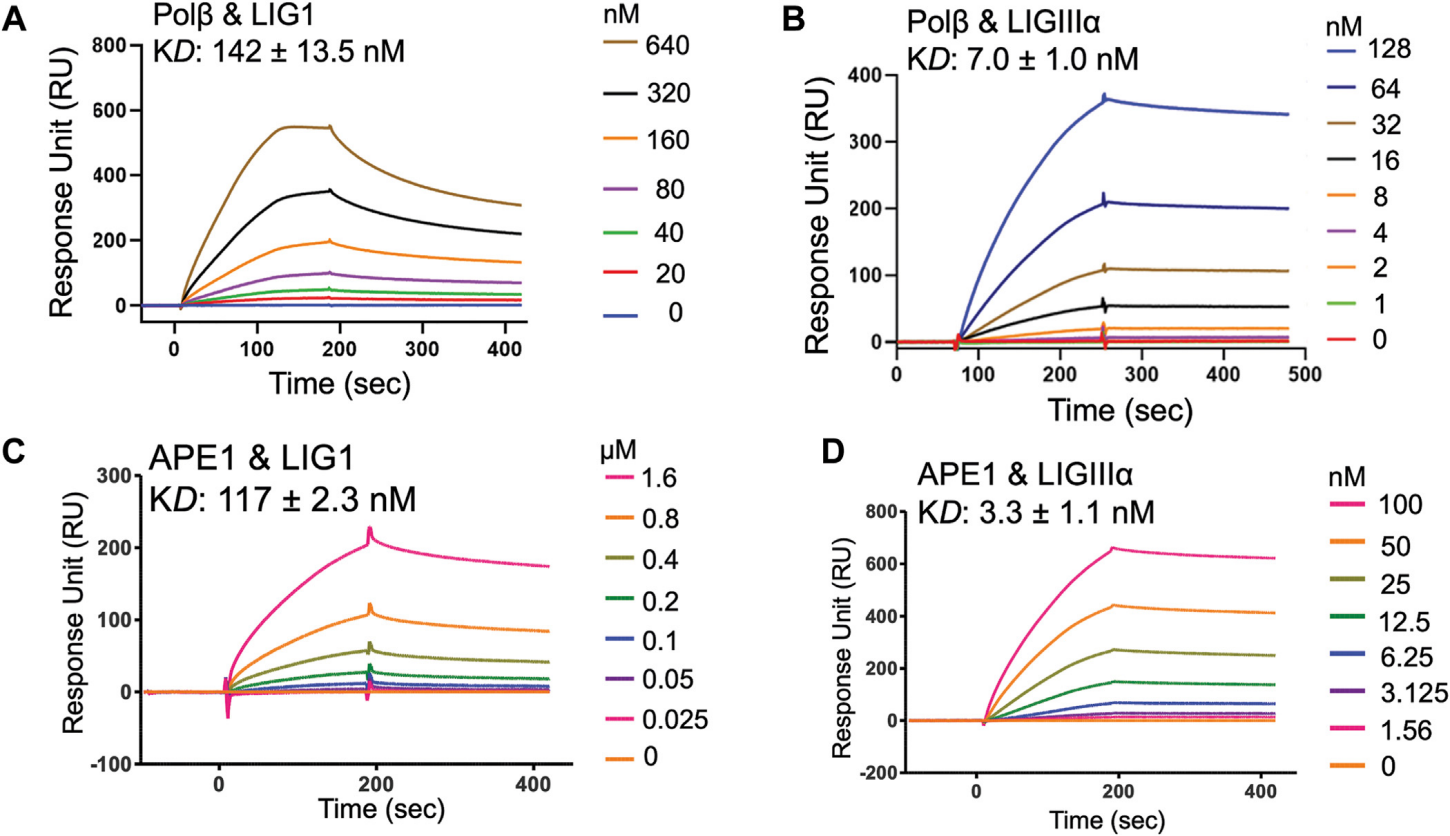
**Protein–protein interaction kinetics of BER ligases.** Protein–protein interactions are shown between polβ and LIG1 (*A*) or LIGIIIα (*B*) and between APE1 and LIG1 (*C*) or LIGIIIα (*D*). The ligand association and dissociation phases are shown for the protein concentration range of DNA ligases on the side of sensorgrams. APE1, apurinic/apyrimidinic-endonuclease 1; BER, base excision repair; LIG, ligase; Pol, polymerase.

**Figure 2:**
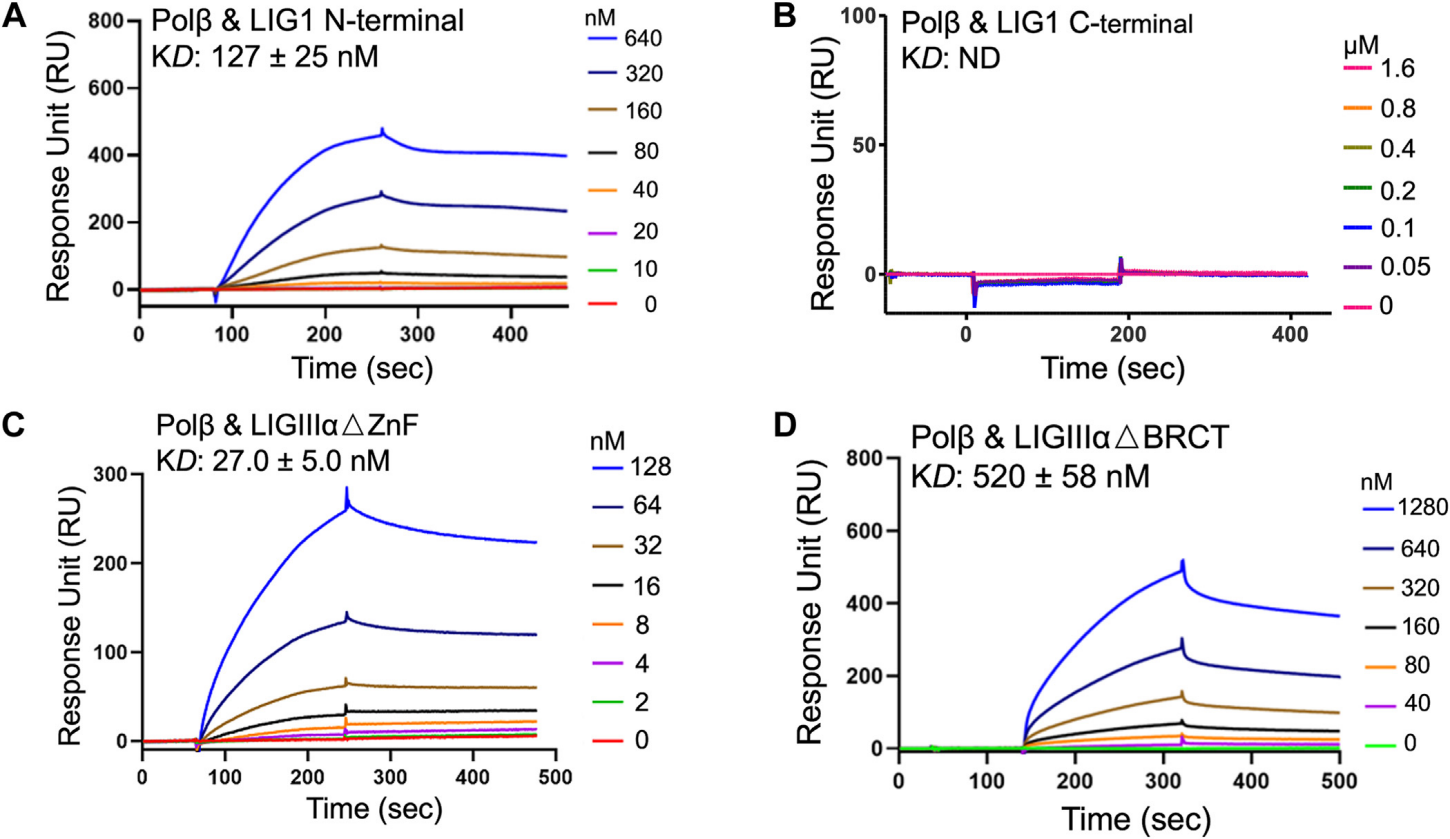
**Protein interaction regions of BER ligases with polβ.** Protein–protein interactions are shown between polβ and LIG1 N-terminal (▵C-terminal mutant, *A*) and LIG1 C-terminal (▵N-terminal mutant, *B*) or LIGIIIα mutants lacking ZnF (▵ZnF mutant, *C*) and BRCT (▵BRCT mutant, *D*). The ligand association and dissociation phases are shown for the protein concentration range of DNA ligases on the side of sensorgrams. BER, base excision repair; BRCT, BRCA1 C terminal; LIG, ligase; Pol, polymerase.

**Figure 3:**
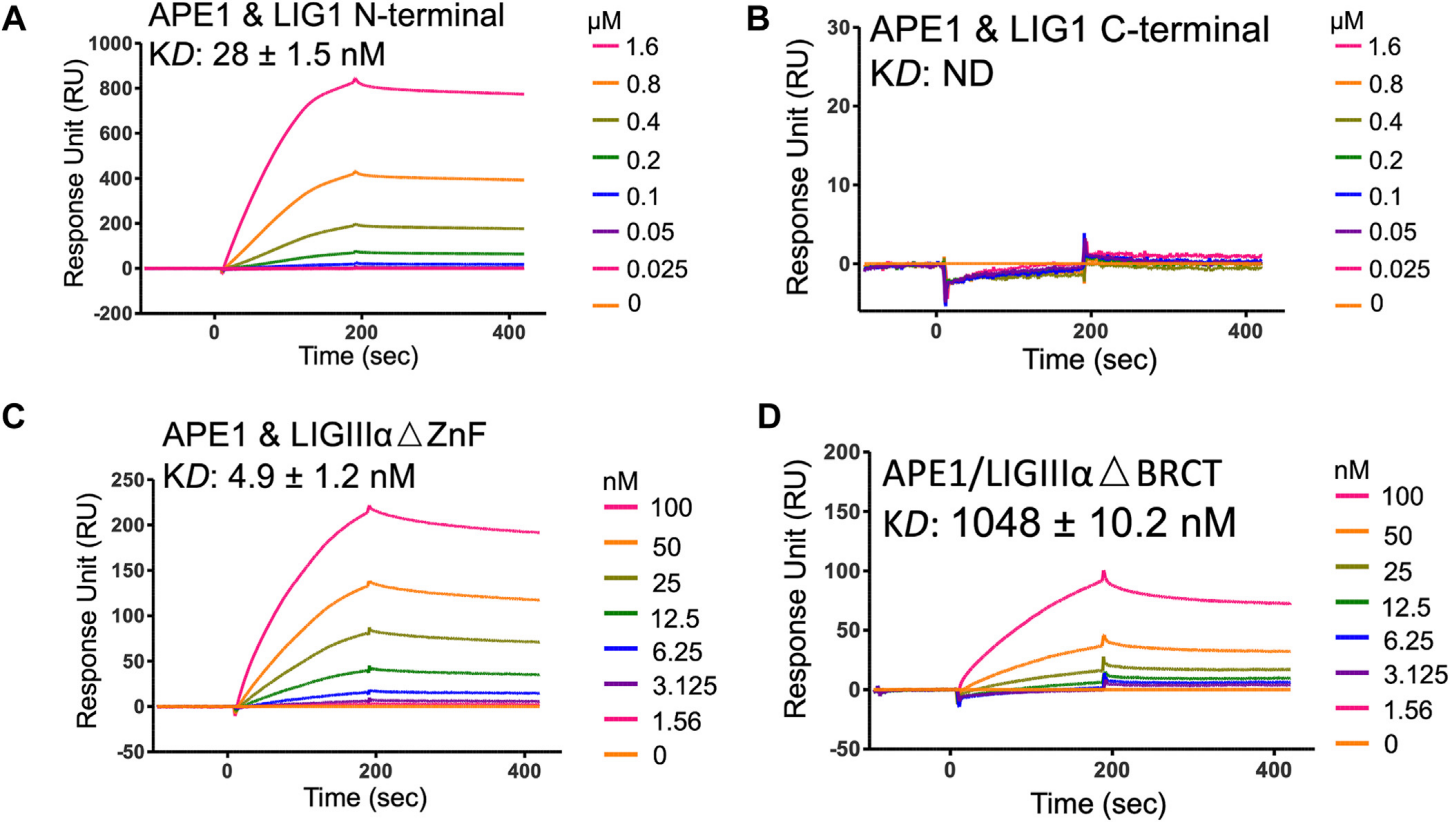
**Protein interaction regions of BER ligases with APE1.** Protein–protein interactions are shown between APE1 and LIG1 N-terminal (▵C-terminal mutant, *A*), LIG1 C-terminal (▵N-terminal mutant, *B*), LIGIIIα mutants lacking ZnF (▵ZnF mutant, *C*), and BRCT (▵BRCT mutant, *D*). The ligand association and dissociation phases are shown for the protein concentration range of DNA ligases on the side of sensorgrams. APE1, apurinic/apyrimidinic-endonuclease 1; BER, base excision repair; BRCT, BRCA1 C terminal; LIG, ligase; ZnF, zinc finger.

Our results showed a ∼20-fold difference in the equilibrium binding constant (K_D_) for polβ interactions with the BER ligases. We demonstrated a stronger interaction between polβ and LIGIIIα (K_D_: 7 nM) than that of polβ and LIG1 (K_D_: 142 nM) for the full-length proteins (Fig. 1, *A* and *B*). Similarly, APE1 interacts with LIGIIIα (K_D_: 3.3 nM) more tightly than LIG1 (K_D_: 117 nM) with a ∼30-fold difference in the K_D_ (Fig. 1, *C* and *D*). We then questioned the regions of the BER ligases that mediate protein interactions with polβ and APE1. For this purpose, we used the truncated mutants of LIG1 and LIGIIIα lacking N- and/or C-terminus regions of the proteins (Figs. 2 and 3). For LIG1, our results demonstrated similar binding affinity of polβ with LIG1 N-terminal region (K_D_: 127 nM) and the full-length protein (K_D_: ∼142 nM). There was no interaction between polβ and LIG1 C-terminal protein, demonstrating that the N-terminal region of LIG1 mediates its interaction with polβ (Fig. 2, *A* and *B*). We showed that the mutant lacking ZnF domain (LIGIIIα▵ZnF) shows weaker interaction with polβ (K_D_: ∼30 nM) than that of the full-length LIGIIIα (K_D_: ∼7 nM). Our measurements demonstrated a significant difference (∼500-fold) for polβ interaction with LIGIIIα lacking BRCT domain with K_D_: 520 nM (Fig. 2, *C* and *D*). Furthermore, using polβ truncated mutants lacking N-terminal and C-terminal domains, we tested protein interactions with the BER ligases (Fig. S2). Our results demonstrated that polβ N-terminal domain mediates its interaction with both LIG1 (K_D_: 237 nM) and LIGIIIα (K_D_: 32 nM). In the presence of polβ C-terminal mutant, we obtained no interaction with LIG1 or significantly reduced (∼500-fold) binding to LIGIIIα in comparison with the full-length proteins (Fig. 1).

We then examined the interaction regions of the BER ligases with APE1 (Fig. 3). Similarly, our results showed that the N-terminal noncatalytic domain of LIG1 mediates the interaction, while the C-terminal catalytic core exhibits no detectable K_D_ (Fig. 3, *A* and *B*). For APE1 and LIGIIIα, we obtained a ∼1000-fold difference in K_D_ between the truncated mutants, LIGIIIα▵ZnF and LIGIIIα▵BRCT, demonstrating that the BRCT domain serves as a platform for APE1 to interact with LIGIIIα (Fig. 3, *C* and *D*). Lastly, we compared the interaction profile of polβ and BER ligases with its well-known partners, XRCC1 and poly (ADP-ribose) polymerase 1 (PARP1). For polβ, our results showed similar binding constants with XRCC1 as we previously reported (27) and PARP1, while PARP1 has ∼10-fold difference in the interaction profile with LIG1 and LIGIIIα (Fig. S3). Overall results demonstrate that the BER ligases interact with the repair proteins, APE1 and polβ that function at earlier steps of the repair pathway, through N-terminal noncatalytic domain of LIG1 and the catalytic core/BRCT domain of LIGIIIα. Furthermore, LIGIIIα is the BER ligase that more tightly binds to both APE1 and polβ (Table S6 and Scheme S2).

### Protein complex formation of BER ligases

Because of tighter interaction between polβ and LIGIIIα as observed by SPR (Fig. 1), we also performed GST pull-down assays where GST-tag polymerase was respectively incubated with his-tag ligase (full-length and truncated mutants) to enable the protein–protein interaction to occur and before being precipitated by GST-binding glutathione beads (Fig. S4). The bound material was then captured in three independent experimental GST pull-down assays and analyzed on SDS-PAGE. Our results showed that the polβ exhibits similar binding to LIGIIIα full-length and truncated mutant lacking ZnF domain, LIGIIIα▵ZnF. We did not observe any binding between polβ and LIGIIIα truncated mutant lacking BRCT domain, LIGIIIα▵BRCT (Fig. S4*A*, lanes 5 and 6 *versus* 7) under the same reaction conditions. In the negative control reactions including GST-tag alone and LIGIIIα (full-length and truncated proteins), we showed no interaction at all (Fig. S4*A*, lanes 8–11), demonstrating that the protein–protein interaction observed was between GST-tag polβ and his-tag LIGIIIα proteins (Fig. S4*A*, lanes 5–7). To validate SPR result showing polβ N-terminal domain harboring dRP-lyase activity is the interaction region with LIGIIIα, we then further performed GST pull-down assays using polβ truncated mutants. Our results showed that the polβ N-terminal domain exhibits similar binding to LIGIIIα full-length and truncated mutant lacking ZnF domain, LIGIIIα▵ZnF (Fig. S4*B*, lanes 5–8). We did not observe a complex formation between polβ C-terminal and LIGIIIα proteins tested (Fig. S4*B*, lanes 1–4). These GST pull-down results further validate the stronger interaction between downstream BER proteins, polβ and LIGIIIα, and the interaction regions of polβ N-terminal and LIGIIIα BRCT domain.

In addition, we performed the protein complex formations of LIGIIIα with polβ and APE1 through the size-exclusion chromatography (SEC). Our SEC analyses demonstrated the BER multiprotein complex including polβ and LIGIIIα in the presence of XRCC1 (Fig. S5*A*). We also showed the protein complex formation of APE1 with LIGIIIα protein containing the catalytic core and BRCT domain when we used truncated mutant lacking ZnF domain, LIGIIIα▵ZnF (Fig. S5*B*). These SEC analyses further validate tight interactions between polβ and APE1 with LIGIIIα and the role of XRCC1 as a scaffolding factor that could be also involved in a stable multiprotein BER complex formation at the downstream steps.

### Impact of BER ligase interactions with polβ on the ligation of gap filling products

**Figure 4:**
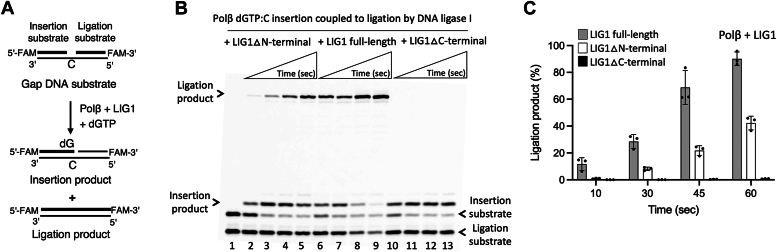
**Ligation of polβ nucleotide insertion products by LIG1.***A*, scheme shows the coupled assay used to test the ligation of polβ dGTP:C insertion products by LIG1 simultaneously in the same reaction mixture. *B*, line 1 is the negative enzyme control of the one nucleotide gap DNA substrate. Lanes 2 to 5, 6 to 9, and 10 to 13 are the ligation of polβ dGTP:C insertion products by LIG1 C-terminal region (▵N-terminal mutant), full-length, and N-terminal region (▵C-terminal mutant), respectively, and correspond to time points of 10, 30, 45, and 60 s. *C*, graph shows time-dependent changes in the amount of ligation products and the data represent the average of three independent experiments ± SD. LIG, ligase; Pol, polymerase.

**Figure 5:**
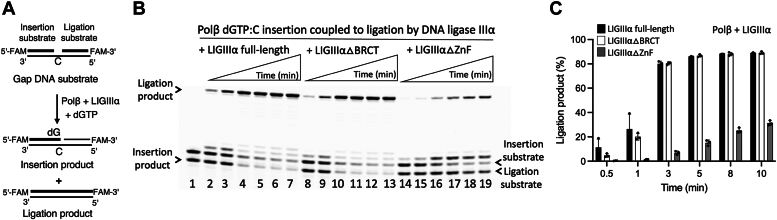
**Ligation of polβ nucleotide insertion products by LIGIIIα.***A*, scheme shows the coupled assay used to test the ligation of polβ dGTP:C insertion products by LIGIIIα simultaneously in the same reaction mixture. *B*, line 1 is the negative enzyme control of the one nucleotide gap DNA substrate. Lanes 2 to 7, 8 to 13, and 14 to 19 are the ligation of polβ dGTP:C insertion products by LIGIIIα full-length, the truncated mutants lacking BRCT (LIGIIIα▵BRCT), and ZnF (LIGIIIα▵ZnF), domains, respectively, and correspond to time points of 0.5, 1, 3, 5, 8, and 10 min. *C*, graph shows time-dependent changes in the amount of ligation products and the data represent the average of three independent experiments ± SD. BRCT, BRCA1 C terminal; LIG, ligase; Pol, polymerase; ZnF, zinc finger.

We next investigated the impact of protein interactions between polβ and the BER ligases on the ligation of polβ nucleotide insertion products *in vitro* (Scheme S1). For this purpose, we performed the coupled assays to measure nick sealing products after polβ gap filling (*i.e*., polβ dGTP insertion into gap DNA containing template base C) by LIG1 or LIGIIIα simultaneously in the same reaction mixture (Figs. 4 and 5).

For polβ and LIG1 (Fig. 4*A*), our results demonstrated the time courses of product formation for polβ dGTP:C insertion and subsequent ligation in the presence of the full-length LIG1 (Fig. 4*B*, lanes 6–9). We obtained a decrease in the products of polβ dGTP:C insertion along with an increase in the formation of nick repair product by LIG1 as a function of reaction time (Fig. 4*C*). In the reactions containing the truncated mutants of LIG1, we observed relatively less efficient nick sealing by LIG1▵N-terminal mutant containing the LIG1 catalytic region only (Fig. 4*B*, lanes 2–5). There were only polβ insertion products and no ligation product in the coupled reaction including LIG1 truncated mutant lacking the catalytic core (LIG1▵C-terminal) as expected (Fig. 4*B*, lanes 10–13). We observed a ∼20-fold difference in the amount of ligation products after polβ nucleotide insertion at earlier time points of the coupled reaction in the absence and presence of the N-terminal region of LIG1 (Fig. 4*C*).

We also investigated the impact of functional interplay between polβ and LIGIIIα in the presence of full-length and the truncated mutants of LIGIIIα (Fig. 5*A*). In the reaction containing LIGIIIα▵BRCT mutant, we obtained an efficient nick sealing of polβ dGTP:C insertion products, which was similar to the ligation products by the full-length protein (Fig. 5*B*, lanes 2–8 and 9–15). On the other hand, in the absence of the ZnF domain (LIGIIIα▵ZnF), we obtained significantly less ligation after polβ dGTP:C insertion in the coupled reaction (Fig. 5*B*, lanes 16–22). There was a ∼20- to 40-fold difference in the amount of ligation products by the effect of ZnF domain (Fig. 5*C*).

### Impact of polβ/DNA ligase interactions on individual BER functions

**Figure 6:**
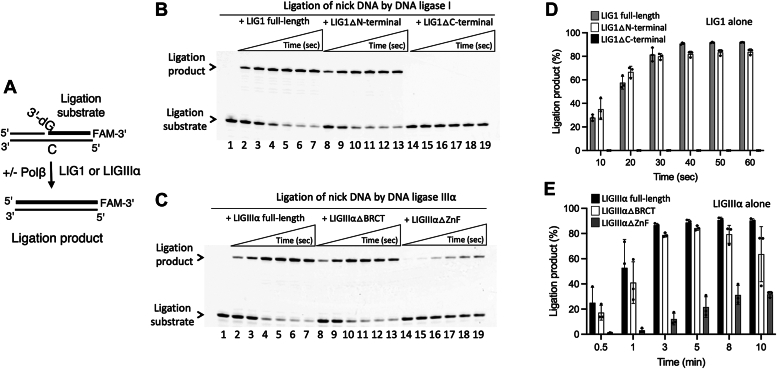
**Ligation of nick DNA by LIG1 and LIGIIIα.***A*, scheme shows the ligation assay used to test the nick sealing efficiency of LIG1 or LIGIIIα in the absence and presence of polβ. *B*, line 1 is the negative enzyme control of the nick DNA substrate. Lanes 2 to 7, 8 to 13, and 14 to 19 are the ligation products in the presence of LIG1 full-length, truncated mutants containing C-terminal (LIG1▵N-terminal), and N-terminal (LIG1▵C-terminal) regions, respectively, and correspond to time points of 10, 20, 30, 40, 50, and 60 s. *C*, line 1 is the negative enzyme control of the nick DNA substrate. Lanes 2 to 7, 8 to 13, and 14 to 19 are the ligation products in the presence of LIGIIIα full-length, truncated mutants lacking BRCT (LIGIIIα▵BRCT) and ZnF (LIGIIIα▵ZnF) domains, respectively, and correspond to time points of 0.5, 1, 3, 5, 8, and 10 min. *D* and *E*, graphs show time-dependent changes in the amount of ligation products and the data represent the average of three independent experiments ± SD. BRCT, BRCA1 C terminal; LIG, ligase; Pol, polymerase; Zn, zinc finger.

We next performed ligation assays using the full-length and truncated mutants of BER ligases to test the ligase activities in the absence and presence of polβ for the nick DNA substrate with preinserted 3′-dG:C (Fig. 6*A*). Our results showed the similar end joining efficiency by LIG1 full-length and truncated mutant containing the catalytic region only, LIG1▵N-terminal (Fig. 6*B*, lanes 2–7 and 8–13). The amount of ligation products showed no significant difference (Fig. 6*D*). We did not observe any ligation product in the presence of LIG1 mutant containing N-terminal noncatalytic region (LIG1▵C-terminal) as expected (Fig. 6*B*, lanes 14–19). Similarly, the ligation efficiency of nick DNA substrate showed no significant difference in the presence of LIGIIIα full-length and truncated mutant lacking BRCT domain, LIGIIIα▵BRCT (Fig. 6*C*, lanes 2–7 and 8–13). However, the nick sealing efficiency was significantly diminished in the absence of ZnF domain when we tested the ligation by LIGIIIα▵ZnF mutant (Fig. 6*C*, lanes 14–19), demonstrating a ∼20- to 40-fold difference in the amount of ligation products at earlier time points of the reaction (Fig. 6*E*).

**Figure 7:**
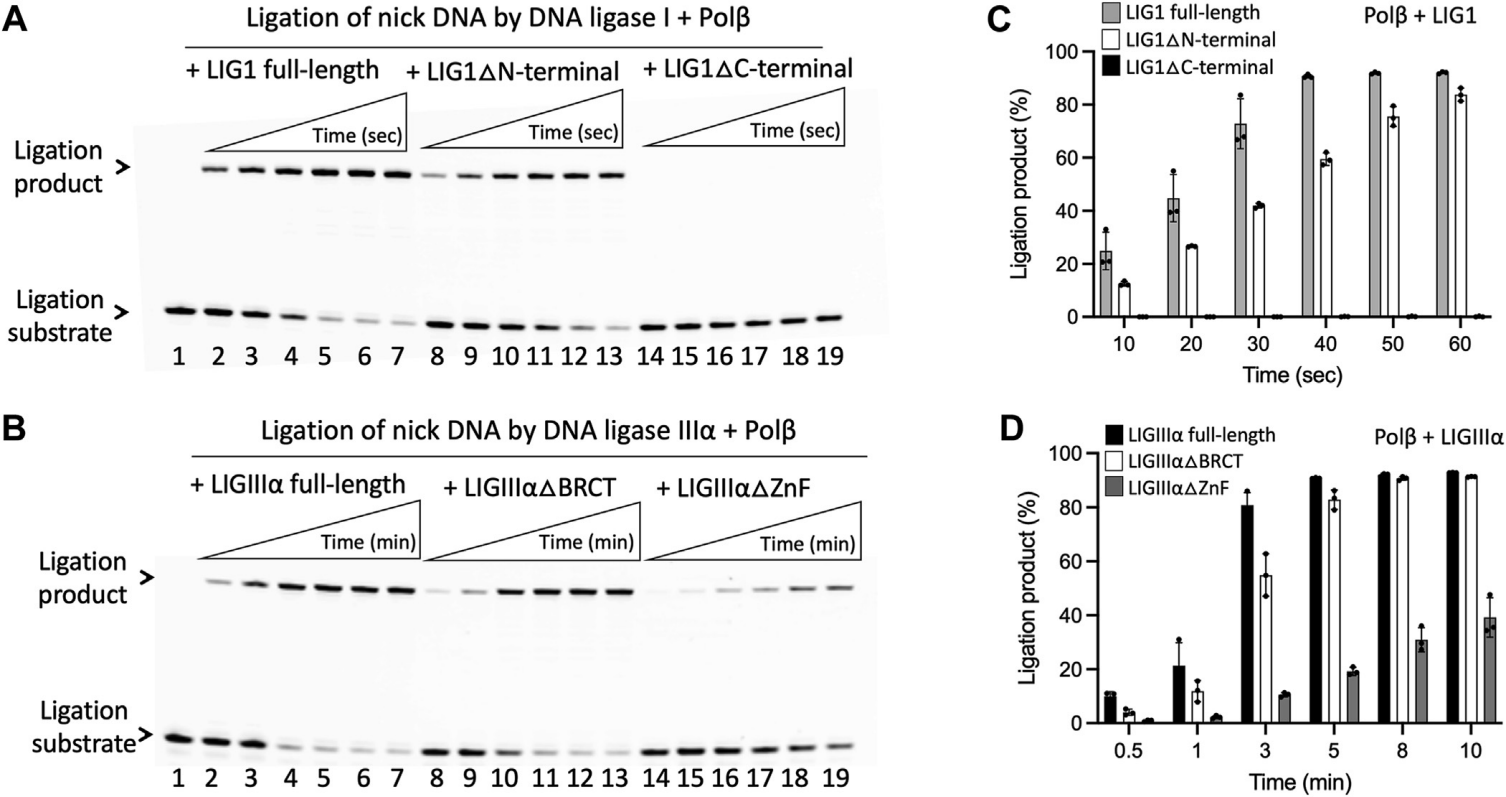
**Ligation of nick DNA by LIG1 and LIGIIIα in the presence of polβ.***A*, line 1 is the negative enzyme control of the nick DNA substrate. Lanes 2 to 7, 8 to 13, and 14 to 19 are the ligation products by LIG1 full-length, truncated mutants containing C-terminal (LIG1▵N-terminal), and N-terminal (LIG1▵C-terminal) regions in the presence of polβ, respectively, and correspond to time points of 10, 20, 30, 40, 50, and 60 s. *B*, line 1 is the negative enzyme control of the nick DNA substrate. Lanes 2 to 7, 8 to 13, and 14 to 19 are the ligation products by LIGIIIα full-length, truncated mutants lacking BRCT (LIGIIIα▵BRCT) and ZnF (LIGIIIα▵ZnF) domains in the presence of polβ, respectively, and correspond to time points of 0.5, 1, 3, 5, 8, and 10 min. *C* and *D*, graphs show time-dependent changes in the amount of ligation products and the data represent the average of three independent experiments ± SD. BRCT, BRCA1 C terminal; LIG, ligase; Pol, polymerase; Zn, zinc finger.

We then repeated the ligation assays in the presence of polβ. Our results demonstrated similar nick sealing efficiency for LIG1 full-length and LIG1▵N-terminal as well as LIGIIIα full-length and LIGIIIα▵BRCT truncated proteins (Fig. 7, *A* and *B*). We did not obtain ligation products in the presence of LIG1▵C-terminal and significantly less ligation products by LIGIIIα▵ZnF (Fig. 7, *C* and *D*). The comparisons of ligation products by LIG1 in the presence of polβ demonstrated no difference for the full-length protein, while we observed a slightly less ligation products by LIG1▵N-terminal at earlier time points (Fig. S6). Similarly, our results showed a ∼2-fold difference in the end joining efficiency of LIGIIIα in the presence of polβ (Fig. S7).

Lastly, we evaluated gap filling activity of polβ in the presence of both BER ligases in the insertion assays using one nucleotide gap DNA substrate. In comparison with a time-dependent increase in dGTP:C insertion products by polβ alone, we obtained very less efficient gap ligation along with the insertion products in the presence of LIG1 and LIGIIIα (Fig. S8, *A*). For the truncated mutants of both DNA ligases, similarly, there was ligation of one nucleotide gap DNA substrate itself by LIG1▵N-terminal and LIGIIIα▵BRCT mutants that we observed in the polβ dGTP:C insertion assays (Fig. S8, *B* and *C*, lanes 2–6), while other mutants yielded no ligation in the insertion assays where we observed polβ products only (Fig. S8, *B* and *C*, lanes 7–11).

As we observed ligation of gap DNA by both ligases in the polβ nucleotide insertion assays including gap DNA substrate, we further tested the efficiency of gap ligation by LIG1 and LIGIIIα using one nucleotide gap DNA substrate with fluorescein labels at both DNA ends that was used the coupled reactions to test the ligation after polβ dGTP insertion simultaneously as described above (Figs. 4 and 5). LIG1 full-length and LIG1▵N-terminal mutant showed very efficient ligation of one nucleotide gap DNA itself and this was similar for LIGIIIα full-length and LIGIIIα▵BRCT mutant (Fig. S9, *A* and *B*). The comparison for the amount of ligation products demonstrated a ∼20- to 60-fold difference between both BER ligases (Fig. S9, *C* and *D*). We confirmed the difference in the size of the products for gap ligation *versus* a nick sealing that can be formed only after polβ correct nucleotide (*i.e*., dGTP:C) insertions in the same reaction mixture (Fig. S10).

### Impact of APE1/DNA ligase interactions on ligation of nick repair intermediates

**Figure 8:**
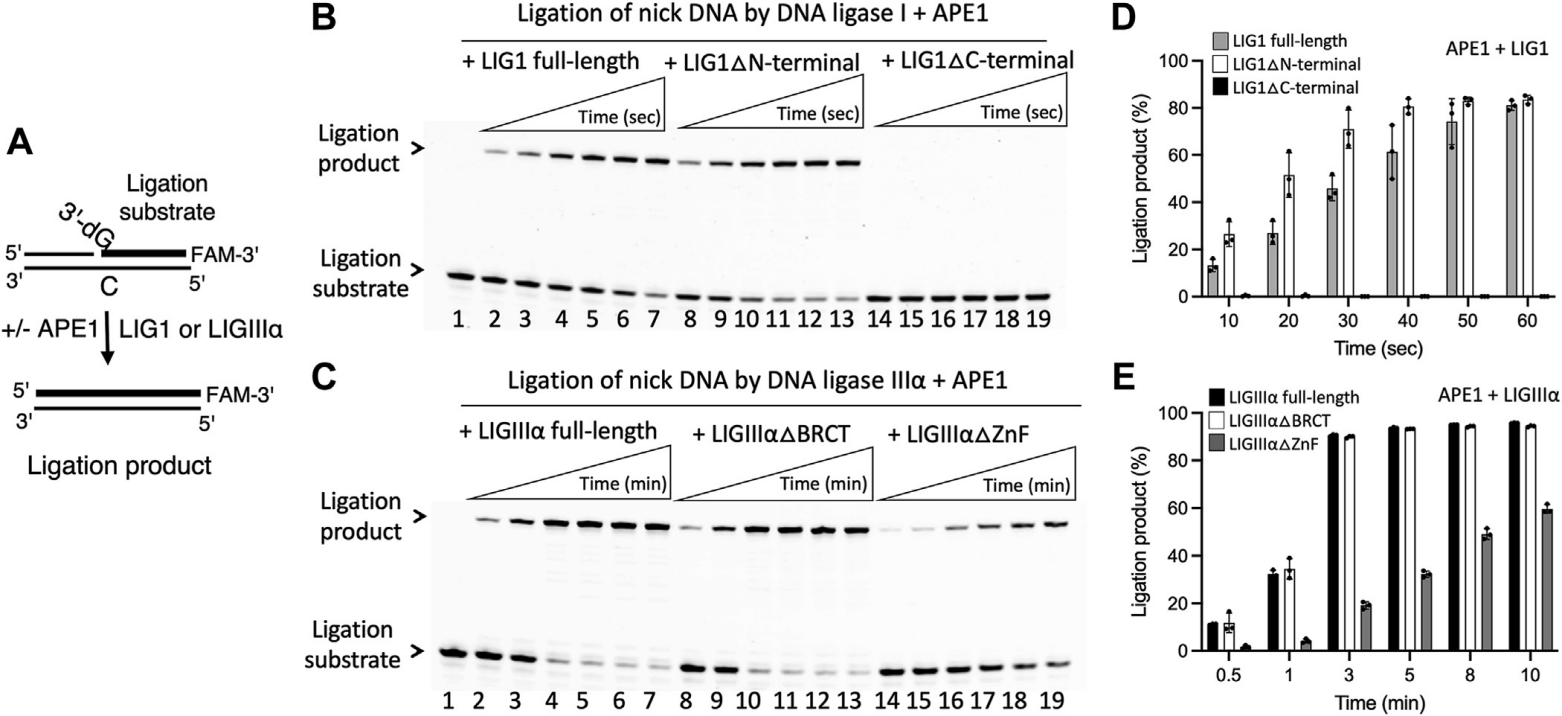
**Ligation of nick DNA by LIG1 and LIGIIIα in the presence of APE1.***A*, scheme shows the ligation assay used to test the nick sealing efficiency of LIG1 or LIGIIIα in the absence and presence of APE1. *B*, line 1 is the negative enzyme control of the nick DNA substrate. Lanes 2 to 7, 8 to 13, and 14 to 19 are the ligation products by LIG1 full-length, truncated mutants containing C-terminal (LIG1▵N-terminal) and N-terminal (LIG1▵C-terminal) regions in the presence of APE1, respectively, and correspond to time points of 10, 20, 30, 40, 50, and 60 s. *C*, line 1 is the negative enzyme control of the nick DNA substrate. Lanes 2 to 7, 8 to 13, and 14 to 19 are the ligation products by LIGIIIα full-length, truncated mutants lacking BRCT (LIGIIIα▵BRCT) and ZnF (LIGIIIα▵ZnF) domains in the presence of APE1, respectively, and correspond to time points of 0.5, 1, 3, 5, 8, and 10 min. *D* and *E*, graphs show time-dependent changes in the amount of ligation products, and the data represent the average of three independent experiments ± SD. APE1, apurinic/apyrimidinic-endonuclease 1; BRCT, BRCA1 C terminal; LIG, ligase; Zn, zinc finger.

We then tested the impact of APE1 on the efficiency of DNA ligation by LIG1 and LIGIIIα using the full-length and truncated mutants (Fig. 8*A*). In the ligation assays with nick DNA substrate containing canonical 3′-dG:C, in the presence of APE1, we observed similar nick sealing efficiency by LIG1 full-length and LIG1▵N-terminal mutant (Fig. 8*B*, lanes 2–13) and by LIGIIIα full-length and LIGIIIα▵BRCT mutant (Fig. 8*C*, lanes 2–13). In the ligation assays with BER ligases and APE1, we did not observe any ligation product in the presence of LIG1▵C-terminal mutant and significantly reduced ligation products by LIGIIIα▵ZnF mutant (Fig. 8, *D* and *E*). The comparison also demonstrated almost same amount of ligation products with and without APE1 (Figs. S6 and S7).

**Figure 9:**
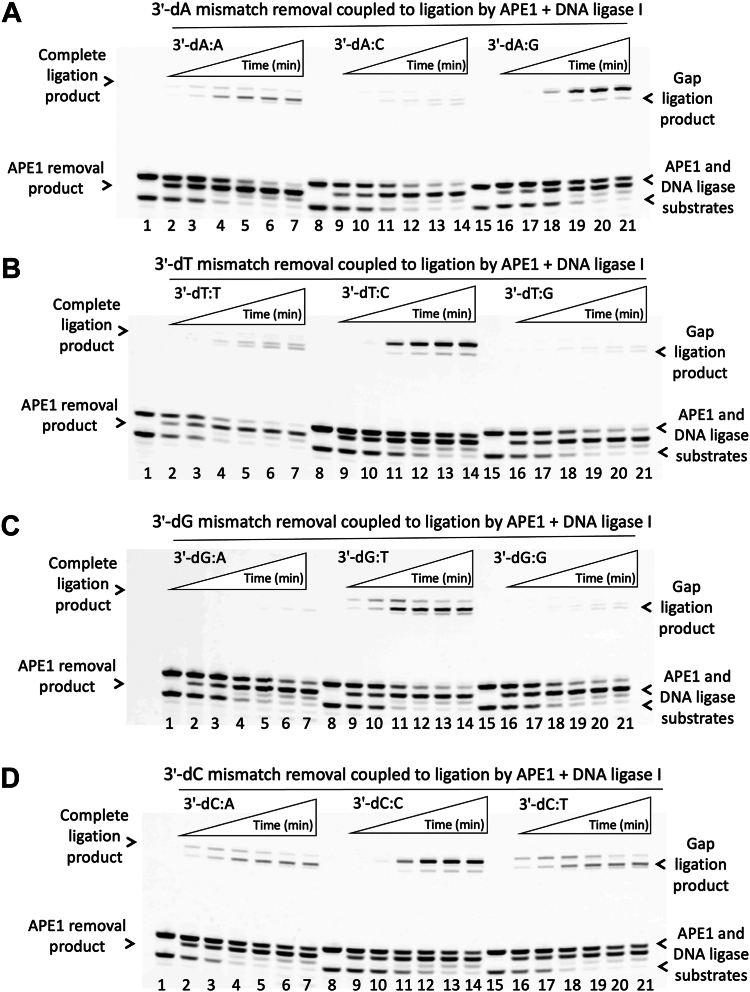
**Mismatch removal coupled to ligation of nick DNA by LIG1 and APE1.***A–D*, lanes 1, 8, and 15 are the negative enzyme controls of the nick DNA substrates containing corresponding mismatches. Lanes 2 to 7, 9 to 14, and 16 to 21 are 3′ mismatch removal by APE1 and ligation products by LIG1 in the presence of nick DNA substrates with 3′-dA:A, 3′-dA:C, 3′-dA:G (*A*); 3′-dT:T, 3′-dT:C, 3′-dT:G (*B*); 3′-dG:A, 3′-dG:T, 3′-dG:G (*C*); and 3′-dC:A, 3′-dC:C, and 3′-dC:T (*D*) mismatches and correspond to time points of 0.5, 1, 3, 5, 8, and 10 min. APE1, apurinic/apyrimidinic-endonuclease 1; LIG, ligase.

**Figure 10:**
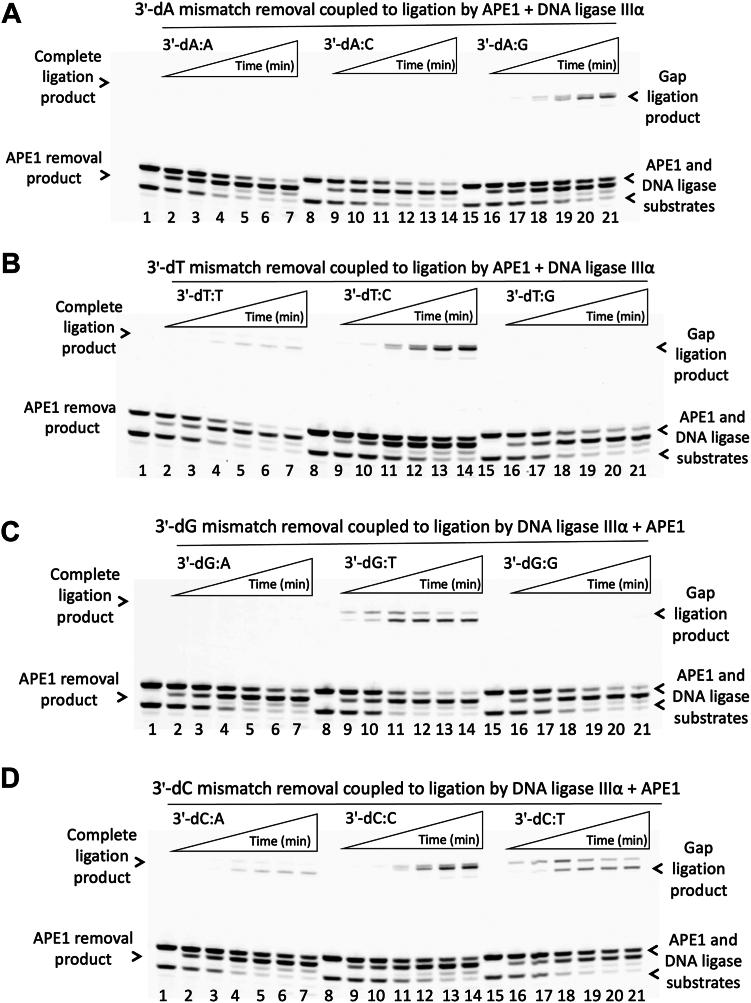
**Mismatch removal coupled to ligation of nick DNA by LIGIIIα and APE1.***A–D*, lanes 1, 8, and 15 are the negative enzyme controls of the nick DNA substrates containing corresponding mismatches. Lanes 2 to 7, 9 to 14, and 16 to 21 are 3′ mismatch removal by APE1 and ligation products by LIGIIIα in the presence of nick DNA substrates with 3′-dA:A, 3′-dA:C, 3′-dA:G (*A*); 3′-dT:T, 3′-dT:C, 3′-dT:G (*B*); 3′-dG:A, 3′-dG:T, 3′-dG:G (*C*); and 3′-dC:A, 3′-dC:C, and 3′-dC:T (*D*) mismatches and correspond to time points of 0.5, 1, 3, 5, 8, and 10 min. APE1, apurinic/apyrimidinic-endonuclease 1; LIG, ligase.

Additionally, we evaluated the impact of APE1/DNA ligase interplay on the processing of the nick repair intermediates with preinserted 3′ mismatches that mimic the products of polβ mismatch insertions. Using the nick substrates containing all possible 12 mismatches at the 3′-end of nick DNA, we performed coupled assays involving both APE1 and DNA ligase to investigate the removal of a mismatched base by APE1 proofreading activity and the ligation of noncanonical nick DNA substrates by LIG1 or LIGIIIα simultaneously in the same reaction (Figs. 9 and 10). In addition, to compare the reaction products of these coupled assays with individual ligase and APE1 activities separately for all 12 possible 3′ mismatches, we evaluated the ligation profile of LIG1 and LIGIIIα individually in the ligation assays as well as the removal of 3′ mismatches by APE1 alone in the exonuclease assays. Our results demonstrated subtle differences in the nick sealing efficiency of LIG1 and LIGIIIα depending on the architecture of the nick DNA substrates containing all 12 possible mismatches (Figs. S11 and S12). Similarly, APE1 exhibits distinct efficiency for the removal of a mismatched base through its proofreading activity from those noncanonical nick substrates with 3′ mismatches (Fig. S13).

When we evaluated APE1 removal coupled to ligation of noncanonical nick DNA substrates by LIG1 or LIGIIIα, our results demonstrated a time-dependent increase in the mismatch removal products (Figs. 9 and 10). Depending on the architecture of 3′ mismatch:template base, these products were appeared along with ligation products by LIG1 and LIGIIIα in the coupled reactions. For LIG1, we obtained the products for both a complete sealing of nick DNA substrate and the ligation of one nucleotide gap after the removal of mismatched base by APE1 in the presence of 3′-dA:A (Fig. 9*A*), 3′-dC:A, and 3′-dC:T (Fig. 9*D*) mismatches. However, we observed more complete ligation products for the nick DNA substrates with 3′-dA:G (Fig. 9*A*, 3′-dT:C (Fig 9*B*), and 3′-dC:C (Fig. 9*D*) mismatches. There was only APE1 removal products we obtained for the nick DNA containing 3′-dA:C (Fig. 9*A*, 3′-dT:G (Fig. 9*B*), and 3′-dG:G (Fig. 9*C*) mismatches.

LIG1 exhibits very efficient ligation of 3′-dT:C, 3′-dC:T, and 3′-dG:T mismatches in contrast to less end joining for 3′-dG:A and 3′-dA:G mismatches (Fig. S11). When compared to the products of coupled assays including APE1 and LIG1, the results demonstrated that exonuclease removal of mismatches was appeared mainly in the coupled reaction including both enzymes although the LIG1 alone shows relatively less efficient ligation for 3′-dA:C (Figs. 9*A* and S11*A*), 3′-dT:G (Figs. 9*B* and S11*B*), and 3′-dG:G (Figs. 9*C* and S11*C*). Particularly, for 3′-dC:A, 3′-dC:T, and 3′-dG:T mismatches, although LIG1 alone shows very efficient nick sealing in the ligation assays, we mainly obtained a time-dependent increase in the ligation of gap repair intermediate following a mismatch removal in the presence of both APE1 and LIG1 in the coupled assays. These results demonstrate a functional interplay between APE1 and LIG1 on the nick repair intermediate to process (proofreading *versus* nick sealing) depending on the nature of noncanonical ends.

The ligation profile of LIGIIIα was relatively less efficient for almost all noncanonical nick substrates and we obtained efficient end joining products only for 3′-dC:T, 3′-dT:C, 3′-dT:G, and 3′-dG:T mismatches (Fig. S12). Interestingly, in the coupled reactions including APE1 and LIGIIIα, the products are both a complete nick sealing and gap ligation after the removal of a mismatched base for 3′-dT:C (Figs. 10*B* and S12*B*), 3′-dG:T (Figs. 10*C* and S12*C*), and 3′-dC:T (Figs. 10*D* and S12*D*). For the rest of the mismatches, we mainly obtained the products of mismatch removal only in the coupled assays including APE1 and LIGIIIα (Fig. 10). These results also demonstrate a difference in the processing of noncanonical nick repair intermediates for the removal of 3′ mismatched base by APE1 when it interacts LIG1 *versus* LIGIIIα at the final steps of the BER pathway.

### DNA-binding affinities of APE1, polβ, and BER ligases

**Figure 11:**
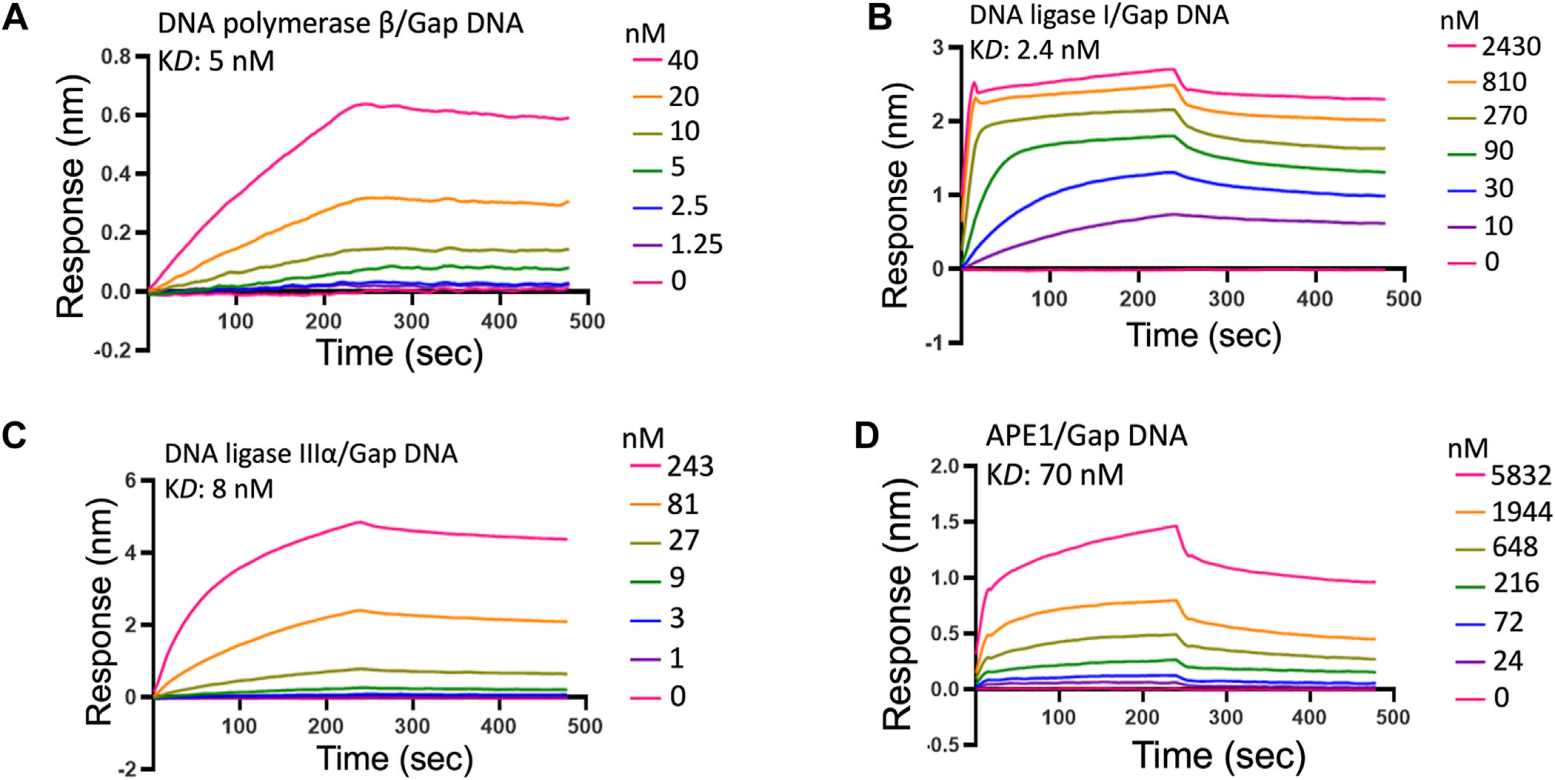
**Gap DNAbinding kinetics of APE1, polβ, and BER ligases.** Gap DNA binding kinetics and the equilibrium binding constants (K_D_) are shown for polβ (*A*), LIG1 (*B*), LIGIIIα (*C*), and APE1 (*D*). Sensorgrams are shown for the concentrations range of the proteins, where one nucleotide gap DNA with a biotin label is immobilized on the streptavidin biosensors. APE1, apurinic/apyrimidinic-endonuclease 1; BER, base excision repair; Pol, polymerase.

**Figure 12:**
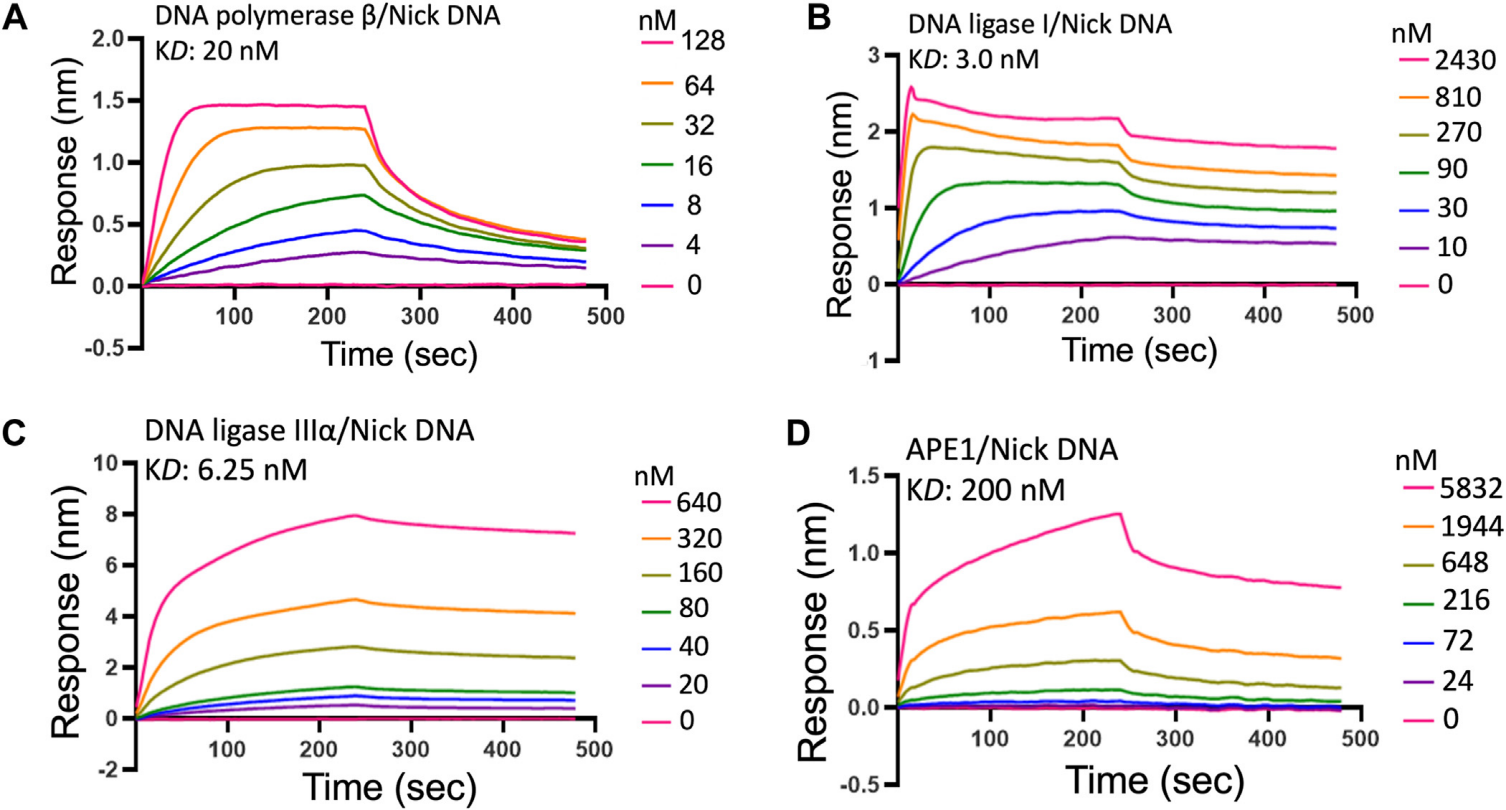
**Nick DNAbinding kinetics of APE1, polβ, and BER ligases.** Nick DNA binding kinetics and the equilibrium binding constants (K_D_) are shown for polβ (*A*), LIG1 (*B*), LIGIIIα (*C*), and APE1 (*D*). Sensorgrams are shown for the concentrations range of the proteins, where the nick DNA with a biotin label is immobilized on the streptavidin biosensor. APE1, apurinic/apyrimidinic-endonuclease 1; BER, base excision repair; Pol, polymerase.

In addition to repair assays with all four major BER proteins, in the present study, we also examined and compared DNA binding affinities of APE1, polβ, LIG1, and LIGIIIα in real time by biolayer interferometry assay (Figs. 11 and 12). For this purpose, we tested one nucleotide gap and nick DNA substrates with 3′-biotin label as these substrates mimic the repair intermediates that APE1, polβ, and both ligases use during BER.

Our results demonstrated that polβ and both BER ligases can bind to one nucleotide gap DNA efficiently and show similar binding affinity with K_D_ values in the range of 3 to 8 nM (Fig. 11, *A–C*). Interestingly, the gap DNA binding efficiency of APE1 was relatively weak (K_D_: 70 nM) demonstrating ∼10-fold difference with other BER proteins (Fig. 11*D*). We did not observe any significant difference in the binding affinity between LIG1 (K_D_: 3 nM) and LIGIIIα (K_D_: ∼7 nM) for nick DNA (Fig. 12, *B* and *C*). Our measurements demonstrated ∼10-fold lower binding with polβ (K_D_: ∼20 nM) than both DNA ligases (Fig. 12*A*). However, APE1 does not show tighter nick DNA binding, and we observed a ∼100-fold difference in the binding affinity (K_D_: 200 nM) in our reaction conditions (Fig. 12*D*).

## Discussion

The BER is a sequential multistep repair process involving a channeling of DNA intermediates in a highly coordinated manner (1, 2, 3). If this normal coordination breaks down, persistent exposure of BER intermediates may trigger harmful nuclease activities, cell death signaling, and can convert into toxic double strand breaks in replicating DNA (7). To date, findings about how BER is orchestrated have emerged from biochemical characterization of the repair enzymes and structure/function analyses of the BER protein/DNA intermediate binary or ternary complexes (9, 10, 11). The proposed models regarding the mechanism of the BER coordination have suggested that first repair enzyme remains bound to the product that is handed off to the next enzyme in the pathway before the first enzyme fully dissociates from the DNA intermediate so that the BER multiprotein complex efficiently executes the substrate-product channeling (12, 13, 14). For example, many DNA glycosylases, that is, the initiating enzyme of BER, bind their abasic site product with higher affinity than the initial base damage substrate, implying that these proteins have evolved to protect cells from the adverse effects of AP sites and facilitate repair by signaling the next enzyme in the pathway. APE1 likewise, after cleaving the DNA backbone, exhibits higher affinity for its incised single-strand break product, coordinating with polβ to carry out efficient 5′-dRP excision and gap filling during the repair response in a process referred to as passing-the-baton or hand off.

BER pathway coordination is mainly mediated by protein–protein interactions and protein-DNA communications between repair proteins as reported using the affinity coprecipitation, two-hybrid analysis, gel filtration, and immunoprecipitation as well as fluorescence titration or polarization-based techniques (46, 47, 48, 49, 50, 51, 52, 53, 54, 55, 56, 57, 58, 59, 60). These studies quantitatively characterized physical protein interactions for the repair enzymes involved in BER such as APE1, polβ, TDP1, PARP1, and LIGIIIα in the absence and presence of BER intermediates and the multiple interacting partners of XRCC1 have been reported in detail as stable complex in cell extracts or using recombinant proteins such as polβ, PNKP, and LIGIIIα (46, 47, 48, 49, 50, 51, 52, 53). Similarly, PARP1 directly interacts with multiple repair enzymes and proteins factors, such as PNKP, polβ, LIGIIIα, and TDP1 through its diverse structural domains to coordinate BER (57, 58, 59, 60). Most recently, single-molecule studies reported the dynamics of coordination between APE1 and polβ, demonstrating that the substrate-product channeling between these BER proteins is dependent on the dissociation kinetics of APE1 and the duration that polβ remains bound near the nick repair intermediate complex consisting of a single nucleotide gap and a 5′-dRP group (61).

Although the early steps of BER have been characterized extensively in detail, the downstream steps in the coordinated process involving DNA ligase activity have been less clearly defined. In the present study, we aimed to interrogate the impact of protein interactions between the BER ligases and the repair proteins, APE1 and polβ, on the repair pathway coordination at the downstream steps during last nick sealing by LIG1 or LIGIIIα. Particularly, the interaction profile of polβ and LIGIIIα remains a significant gap in our knowledge, although extensive biochemical and structural characterization of XRCC1/LIGIIIα interaction through BRCT domains of both proteins have been reported (45). The direct binding partners of LIGIIIα, through BRCT domain, have been reported as the NEIL1, NEIL2, PNKP, PARP2, and TDP1 (53, 55, 56, 58, 59, 60). Regarding LIG1, the domain mapping and thermodynamics studies have shown that polβ can physically interact through the N-terminal regions of both proteins and specific interaction was obtained in a multiprotein BER complex from bovine testis (38, 39). Furthermore, DNA-free stable large BER multiprotein complexes including PARP1, LIGIIIα, and XRCC1 have been isolated from cell extracts and mammalian tissues depending on protein–protein interactions, while several important BER factors such as DNA glycosylases, APE1, and flap endonuclease 1 were not recovered on those complexes (62, 63, 64, 65, 66, 67, 68, 69). This could be due to the individual factors within the large complex have sufficient affinity for one another for the recruitment of a stable multiprotein complex in the absence of a DNA lesion or a large BER protein complex could reflect a postlesion repair where a multiprotein complex was disassembled after DNA lesion is repaired (11).

In the present study, we have identified the regions that mediate LIGIIIα and LIG1 protein interactions with APE1 and polβ. Our findings demonstrated that LIGIIIα is the ligase that binds to both BER proteins more tightly, suggesting that APE1/polβ/LIGIIIα could form a stronger multiprotein complex during short-patch BER subpathway, where a single DNA base damage is repaired as also demonstrated in repair reconstituted assays *in vitro* (12). We showed that the N-terminal noncatalytic domain of LIG1 that interacts with DNA replication proteins such as PCNA, RFA, and RFC is also the region for its interaction with APE1 and polβ (Scheme S2). Similarly, the BRCT domain provides a platform for LIGIIIα interaction with APE1 and polβ in addition to its well-known interacting partner XRCC1 (Scheme S2). Furthermore, our results revealed the functional impact of these interactions on the repair pathway coordination during gap filling and subsequent nick sealing steps at the downstream steps. We showed that the N-terminal domain of LIG1 is required for the efficient ligation of polβ nucleotide insertion products. Although this region does not contain catalytic residues, our results suggest that this noncatalytic region could play a role for efficient hand off resulting nick product to DNA ligase after polβ gap filling at the final steps. However, our results with LIGIIIα lacking BRCT domain showed same ligation efficiency with the full-length protein although we observed a difference in the interaction profile with polβ. This could be due to the presence of the ZnF and DBD domains that function cooperatively as DNA binding module for nick sealing as reported (43, 44). Indeed, we obtained drastically less efficient ligation of polβ nucleotide insertion products in the presence of the LIGIIIα lacking N-terminal ZnF domain, which supports the role of this region as DNA nick sensor that distinguishes LIGIIIα from the other mammalian DNA ligases. Our findings further demonstrate its importance for sealing of nick repair products after polβ-mediated nucleotide insertions during BER. In addition to polβ/DNA ligase functional interplay, our study also revealed a functional interplay between proofreading role of APE1 for the removal of mismatches and ligation of nick repair intermediates containing noncanonical ends by BER ligases. This APE1/DNA ligase interplay could provide a fidelity check point at the final steps for efficient BER (Scheme S2). We also showed that LIG1 and LIGIIIα can attempt ligating gap DNA after the removal of 3′ mismatches by APE1 and both ligases can bind to gap DNA as efficient as nick DNA. As shown extensively in biochemical and cell studies (67), 30% of human tumors such as lung, gastric, colorectal, and prostate cancer express polβ variants carrying single amino acid substitutions, which lead to an aberrant activity *in vitro* such as a diminished gap filling activity (E295K). We suggest that in case of any deviations in gap filling function of polβ during BER, such as such polβ cancer-associated variants possessing aberrant BER function, the gap ligation by BER ligases can lead to the faulty repair events and formation of single deletion mutagenesis products, which in turn could lead to an interruption in the proper BER repair pathway coordination at the downstream steps.

It has been known that XRCC1, a nonenzymatic scaffold protein, assembles multiprotein complexes (15). In our previous report (27), we also demonstrated that the polβ/XRCC1 complex enhances the handoff of nicked repair products to the final ligation step and XRCC1 cancer-associated (P161L, R194W, R280H, R399Q, Y576S) and cerebellar ataxia–related (K431N) variants impact this channeling process distinctly depending on the nature of the mutation. Furthermore, in our previous study (27), we demonstrated that XRCC1 has a stabilizing effect on the formation of polβ/dNTP/gap DNA and LIGIIIα/ATP/nick DNA catalytic ternary complexes. In the present study, we also showed protein complex formations between polβ and LIGIIIα in the presence of XRCC1 as well as between APE1 and LIGIIIα through SEC, demonstrating that tight interactions mediated by LIGIIIα with the core BER proteins could facilitate processivity of BER reactions between repair proteins during damage processing in a multiprotein assembly.

Overall, these findings demonstrate how multiprotein BER complex containing APE1, polβ, and LIG1/LIGIIIα coordinate during the processing of gap and nick repair intermediates at the final steps. Further single-molecule studies in real time are needed to visualize the dynamics of polβ/DNA ligase interplay at the downstream steps. Furthermore, the cryo-EM studies of large multiprotein BER assemblies are required to elucidate the complex interactions between downstream proteins to gain an insight into the precise three-dimensional architecture of the BER interaction network. Elucidating the interacting regions of protein–protein interactions within the BER repair complex is of high importance for exploiting as novel chemotherapeutic targets (68, 69).

## Experimental procedures

### Protein purifications

DNA pol β (pGEX-6p-1) protein (1–335 amino acids) was overexpressed in BL21(DE3) *Escherichia coli* (*E. coli*) cells in lysogeny broth media at 37 °C for 8 h and induced with 0.5 mM isopropyl-B-D-1-thiogalactopyranoside. The cells were then grown overnight at 16 °C. After cell lysis at 4 °C by sonication in the lysis buffer containing 1× PBS (pH 7.3), 200 mM NaCl, 1 mM dithiothreitol (DTT), and cOmplete protease inhibitor cocktail, the lysate was pelleted at 16,000× rpm for 1 h and then clarified by centrifugation and filtration. The supernatant was loaded on GSTrap HP column and purified with the elution buffer containing 50 mM Tris–HCl (pH 8.0) and 10 mM reduced GSH. To cleave the GST-tag, the recombinant polβ protein was incubated with PreScission Protease for 16 h at 4 °C in the buffer containing 1× PBS (pH 7.3), 200 mM NaCl, and 1 mM DTT. After the cleavage, the polβ protein was subsequently passed through GSTrap HP column, and the protein without the GST-tag was then further purified by loading onto Superdex 200 Increase 10/300 column in the buffer containing 50 mM Tris–HCl (pH 7.5), 400 mM NaCl, and 5% glycerol. Polβ truncated mutants C-terminal (92–335 amino acids) and N-terminal (1–92 amino acids) proteins were purified similarly with the full-length protein. DNA LIGIIIα (pET-24b) protein for the full-length (1–922 amino acids) was overexpressed in BL21(DE3) *E. coli* cells in lysogeny broth media at 37 °C for 8 h and induced with 0.5 mM IPTG. The cells were harvested, lysed at 4 °C, and then clarified as described above. The supernatant was loaded onto HisTrap HP column and purified with an increasing imidazole gradient (0–300 mM) elution at 4 °C. The collected fractions were then further purified by Superdex 200 Increase 10/300 column in the buffer containing 50 mM Tris–HCl (pH 7.0), 500 mM NaCl, 5% glycerol, and 1 mM DTT. LIGIIIα truncated proteins LIGIIIα▵ZnF (170–922 amino acids) and LIGIIIα▵BRCT (1–755 amino acids) were purified similarly with the full-length protein. LIG1 (pET-24b) full-length (1–919 amino acids) protein was overexpressed in BL21(DE3) *E. coli* cells and the cells were harvested, lysed at 4 °C, and then clarified as described above. The supernatant was loaded on HisTrap HP column and purified with an increasing imidazole gradient (0–300 mM) elution at 4 °C. The collected fractions were then subsequently loaded on HiTrap Heparin column with a linear gradient of NaCl up to 1 M. His-tag LIG1 proteins were then further purified by Superdex 200 Increase 10/300 column in the buffer containing 50 mM Tris–HCl (pH 7.0), 500 mM NaCl, 5% glycerol, and 1 mM DTT. LIG1 truncated mutants LIG1▵C-terminal (1–262 amino acids) and LIG1▵N-terminal (262–919 amino acids) were purified similarly with the full-length protein. APE1 protein (pET-24b) was overexpressed in BL21(DE3) *E. coli* cells and the cells were harvested, lysed at 4 °C, and the supernatant was loaded onto HisTrap HP column as described above. His-tag APE1 protein was purified with an increasing imidazole gradient (0–300 mM) elution and then loaded onto HiTrap Heparin column to further purify as a linear gradient of NaCl up to 1 M, and then finally loaded on Superdex 200 Increase 10/300 column in the buffer containing 50 mM Tris–HCl (pH 7.0), 500 mM NaCl, 5% glycerol, and 1 mM DTT. XRCC1 and PARP1 (pET-24b) proteins were overexpressed in BL21(DE3) *E. coli*, and the cells were harvested, lysed at 4 °C, and then clarified as described above. The supernatant was loaded on HisTrap HP column, purified with an increasing imidazole gradient (0–300 mM) elution, and then subsequently loaded on HiTrap Heparin column with a linear gradient of NaCl up to 1 M. His-tag XRCC1 and PARP1 proteins were then further purified by Superdex 200 Increase 10/300 column in the buffer containing 50 mM Tris–HCl (pH 7.0), 500 mM NaCl, 5% glycerol, and 1 mM DTT. All proteins used in this study were dialyzed against storage buffer containing 25 mM Tris–HCl (pH 7.4), 100 mM KCl, 1 mM tris 2-carboxyethyl phosphine, and 10% glycerol, concentrated, frozen in liquid nitrogen, and stored at −80 °C in aliquots. The final purity of all proteins used in this study was evaluated by 10% SDS-PAGE analyses (Fig. S1).

### SPR assay for protein–protein interaction measurements

We analyzed the protein–protein interactions of LIG1 and LIGIIIα with APE1, polβ, XRCC1, and PARP1 by SPR. The experiments were carried out using Biacore X-100 (Cytiva) at 25 °C in real time. One flow cell of the CM5 sensor chip was activated with a 1:1 mixture of 0.2 M EDC and 0.05 M NHS in water, and then the interacting protein partner was injected over the flow cell in 10 mM sodium acetate acetate, at pH 4.0 at a flow rate of 10 μl/min. The binding sites were blocked using 1 M ethanolamine (pH 8.5) and protein partners ranging in the concentrations as indicated in the Figure legends were then injected for 2 min at a flow rate of 30 μl/min in the running buffer containing 10 mM Hepes (pH 7.4), 150 mM NaCl, 3 mM EDTA, and 0.005% (v/v) surfactant P20. After a dissociation phase for 3 to 4 min, 10 mM glycine-HCl (pH 2.0) was injected for 30 s to regenerate the chip surface. Nonspecific binding to a blank flow cell was subtracted to obtain corrected sensorgrams. To obtain *K*_*D*_, the data were analyzed using BIAevaluation software version 2.0.1 and fitted to a 1:1 (Langmuir) binding model.

### DNA-binding measurements by biolayer interferometry assay

We analyzed DNA-binding kinetics of APE1, polβ, LIG1, and LIGIIIα by biolayer interferometry assays in real time using the Octet QKe (Fortebio). DNA-binding kinetics was performed using one nucleotide gap and nick DNA substrates with 3′-biotin label (Table S1). Streptavidin (SA) biosensors were used to attach the biotin labeled DNA. For APE1, polβ, and LIGIIIα, the SA biosensors were hydrated at 20 °C for 20 min in the kinetics buffer containing 20 mM Hepes (pH 7.4), 200 mM NaCl, 0.5% bovine serum albumin (BSA), and 0.05% Tween 20. For LIG1, the SA biosensors were hydrated at 20 °C for 20 min in the buffer containing 50 mM Tris–HCl (pH 7.5), 100 mM KCl, and 1 mM DTT. The sensors were then immersed in DNA (40 nM) in the buffer for 300 s. After recording an initial baseline in the buffer for 60 s, the sensors with DNA were exposed to the concentration range of polβ, APE1, LIG1, or LIGIIIα for gap and nick DNA binding at the concentration range as indicated in the Figure legends. DNA binding was performed for 240 s association and then in the buffer for 240 s dissociation. In all measurements, the affinity constants (K_D_), the association (k_on_), and dissociation (k_off_) rates were calculated using the ForteBio Data Analysis software with 1:1 binding model. The association rate = k_on_ [ligand][analyte] and the dissociation rate = k_off_ [ligand-analyte]. At equilibrium, forward and reverse rates are equal. All images were drawn using GraphPad Prism 7.

### GST pull-down assays

GST pull-down assays were performed to validate the protein–protein interactions of polβ and LIGIIIα. Briefly, his-tag LIGIIIα protein (5 μM) was incubated with GST-tag polβ (5 μM) in the reaction mixture containing 50 mM Tris–HCl (pH 7.5), 100 mM NaCl, and 1 mM DTT at 4 °C for 2 h. The proteins were then mixed with 20 μl of glutathione sepharose beads with constant rotation at 4 °C for 2 h. The beads were washed three times with the 1 ml assay buffer and then by the elution buffer containing 50 mM Tris–HCl (pH 8.0) and 10 mM reduced GSH. The eluted protein samples were analyzed on SDS-PAGE, and the gels were scanned by AI680 (Amersham RGB). GST pull-down assays were performed similarly for LIGIIIα truncated proteins LIGIIIα▵ZnF (170–922 amino acids) and LIGIIIα▵BRCT (1–755 amino acids).

### SEC of BER protein complexes

The BER protein complexes of APE1/LIGIIIα and polβ/XRCC1/LIGIIIα were obtained using SEC. Briefly, the proteins were prepared at equimolar 1:1 or 1:2.5 ratio in the buffer containing 50 mM Tris (pH 8.0), 200 mM NaCl, and 1 mM DTT. The protein complexes were incubated for 2 h on ice prior to SEC analysis and were analyzed using Superdex 200 Increase GL 10/30 column in the same buffer, where the BER protein complexes were made. The fractions corresponding to the peaks were collected and analyzed on 12% SDS-PAGE for shifts in elution volumes, and the gels were scanned by AI680 (Amersham RGB).

### Polβ nucleotide insertion assays

One nucleotide gap DNA substrates with template C was used (Table S2) to test polβ dGTP insertion in the absence and presence of LIG1 or LIGIIIα (Scheme S1). The reaction mixture contains 50 mM Tris–HCl (pH 7.5), 100 mM KCl, 10 mM MgCl_2_, 1 mM ATP, 1 mM DTT, 100 μg ml^−1^ BSA, 1% glycerol, dGTP (100 μM), and DNA substrate (500 nM) in the final volume of 10 μl. The reaction was initiated by the addition of polβ alone (10 nM) or after its preincubation with LIG1 or LIGIIIα (10 nM). The reaction mixtures were incubated at 37 °C for the time points as indicated in the figure legends. The reaction products were then mixed with an equal amount of gel loading buffer containing 95% formamide, 20 mM EDTA, 0.02% bromophenol blue, and 0.02% xylene cyanol and separated by electrophoresis on 18% Urea-PAGE gel. The gels were finally scanned with a Typhoon PhosphorImager (Amersham Typhoon RGB), and the data were analyzed using ImageQuant software. The insertion assays were performed similarly for the full-length and truncated mutants of LIG1 (▵C-terminal and ▵N-terminal) or LIGIIIα (▵BRCT and ▵ZnF).

### Polβ nucleotide insertion coupled to DNA ligation assays

One nucleotide gap DNA substrate with template C (Table S2) was used to test the ligation of polβ dGTP:C insertion products by LIG1 or LIGIIIα (Scheme S1). The reaction mixture contains 50 mM Tris–HCl (pH 7.5), 100 mM KCl, 10 mM MgCl_2_, 1 mM ATP, 1 mM DTT, 100 μg ml^−1^ BSA, 1% glycerol, dGTP (100 μM), and DNA substrate (500 nM) in the final volume of 10 μl. The reaction was initiated by the addition of the preincubated polβ/DNA ligase protein complex (100 nM) and incubated at 37 °C for the time points as indicated in the figure legends. The reaction products were then mixed with an equal amount of gel loading buffer, separated by electrophoresis on 18% Urea-PAGE gel, and the data were analyzed using ImageQuant software as described above. The coupled assays were performed similarly for the full-length and truncated mutants of LIG1 (▵C-terminal and ▵N-terminal) or LIGIIIα (▵BRCT and ▵ZnF).

### DNA ligation assays

Nick DNA substrates with a canonical 3′-dG:C (Table S3) was used to test the nick sealing efficiency of LIG1 or LIGIIIα in the absence and presence of polβ or APE1 (Scheme S1). Nick DNA substrates with all possible noncanonical 12 mismatches (Table S3) were used to test the nick sealing efficiency of LIG1 or LIGIIIα (Scheme S1). The reaction mixture contains 50 mM Tris–HCl (pH 7.5), 100 mM KCl, 10 mM MgCl_2_, 1 mM ATP, 1 mM DTT, 100 μg ml^−1^ BSA, 1% glycerol, and DNA substrate (500 nM) in the final volume of 10 μl. The reaction was initiated by the addition of LIG1 or LIGIIIα (100 nM) alone or after its preincubation with polβ or APE1 (100 nM). The reaction mixtures were incubated at 37 °C for the time points as indicated in the figure legends. The reaction products were then mixed with an equal amount of gel loading buffer, separated by electrophoresis on 18% Urea-PAGE gel, and the data were analyzed using ImageQuant software as described above. The ligation assays were performed similarly for the full-length and truncated mutants of LIG1 (▵C-terminal and ▵N-terminal) or LIGIIIα (▵BRCT and ▵ZnF).

### APE1 assays

Nick DNA substrates with preinserted 3′ mismatches were used (Table S4) to test APE1 exonuclease activity for the removal of 3′ mismatched base in the assays including APE1 alone (Scheme S1). Nick DNA substrates with preinserted 3′ mismatches (Table S5) were used to test the ligation after APE1 removal by LIG1 or LIGIIIα in the coupled assays including both APE1 and DNA ligases (Scheme S1). The reaction mixture contains 50 mM Tris–HCl (pH 7.5), 100 mM KCl, 10 mM MgCl_2_, 1 mM ATP, 1 mM DTT, 100 μg ml^−1^ BSA, 1% glycerol, and DNA substrate (500 nM) in the final volume of 10 μl. The reaction was initiated by the addition of the preincubated APE1/DNA ligase protein complex (100 nM) and incubated at 37 °C for the time points as indicated in the figure legends. The reaction products were then mixed with an equal amount of gel loading buffer, separated by electrophoresis on 18% Urea-PAGE gel, and the data were analyzed using ImageQuant software as described above.

## Data availability

All data are contained within the article. Further information and requests of materials used in this research should be directed to M. Ç. (caglayanm@ufl.edu).

## Supporting information

This article contains supporting information.

## Conflict of interest

The authors declare that they have no conflicts of interest with the contents of this article.

## Supporting information

Supplemental Figures S1–S13, Tables S1–S6, and Schemes S1 and S2
