# Supplementary material for: Impact of DNA ligase 1 and IIIα interactions with APE1 and polβ on the efficiency of base excision repair pathway at the downstream steps
Source: J Biol Chem. 2024 May 7;300(6):107355. doi: 10.1016/j.jbc.2024.107355 (PMC11176775; doi:10.1016/j.jbc.2024.107355)
Supplement: Supplemental Figures S1–S13, Tables S1–S6, and Schemes S1 and S2 [file mmc1.pdf]

Impact of DNA ligase 1 and IIIα interactions with APE1 and polβ on the efficiency of base excision repair pathway at the downstream steps

Danah Almohdar, David Murcia, Qun Tang, Abigail Ortiz, Ernesto Martinez, Tanay Parwal, Pradnya Kamble, Melike Çağlayan*

Department of Biochemistry and Molecular Biology, University of Florida, Gainesville, FL 32610, USA

*To whom correspondence should be addressed. Tel.: +1 352-294-8383; Email: caglayanm@ufl.edu

Supplementary Information

Supplementary Figures 1-13

Supplementary Schemes 1-2

Supplementary Tables 1-6

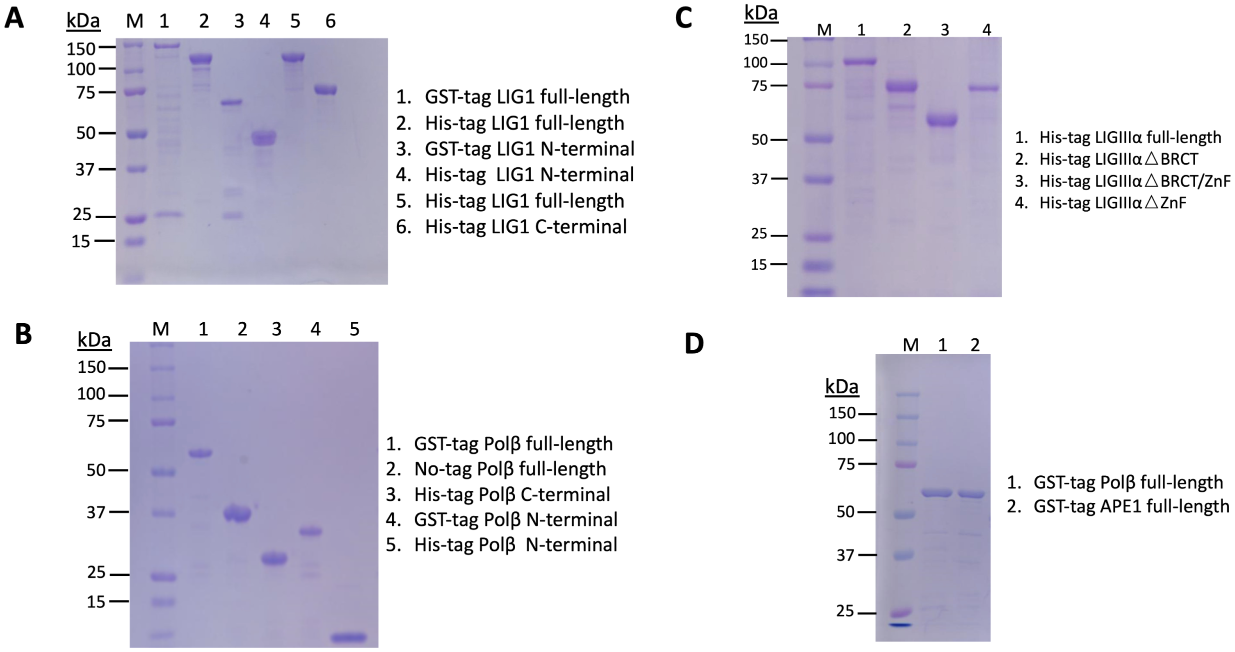

Supplementary Figure 1. **BER proteins used in the study.** Final purity of BER proteins used in the study are shown for LIG1 (**A**), polβ (**B**), LIGIIIα (**C**), and APE1 (**D**). M represents a Precision Plus Protein Dual Color Standard (10-250 kDa).

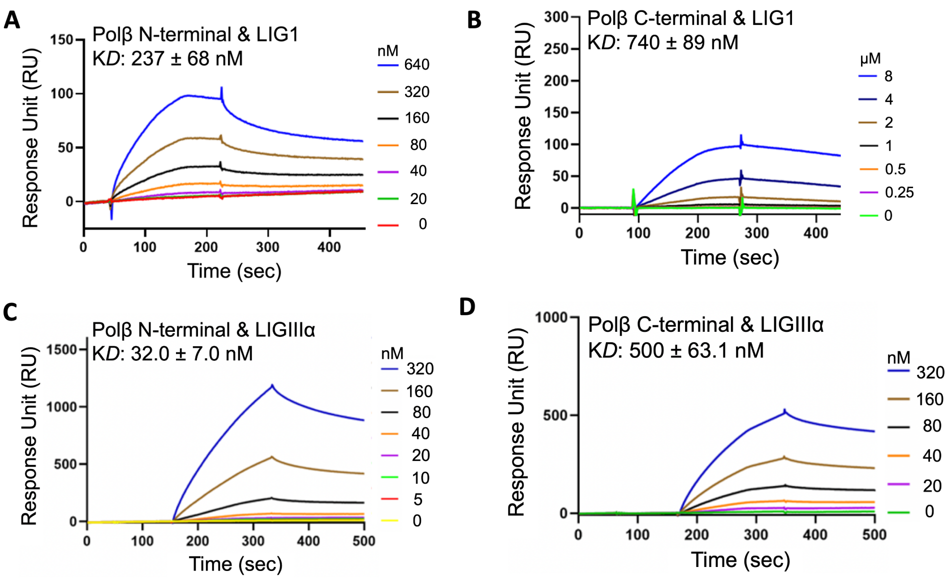

Supplementary Figure 2. **Interaction profile of LIG1 and LIGIIIα with polβ truncated proteins.** Protein-protein interaction measurements in real-time are shown between polβ N-terminal or polβ C-terminal and LIG1 (**A-B**) and LIGIIIα (**C-D**).

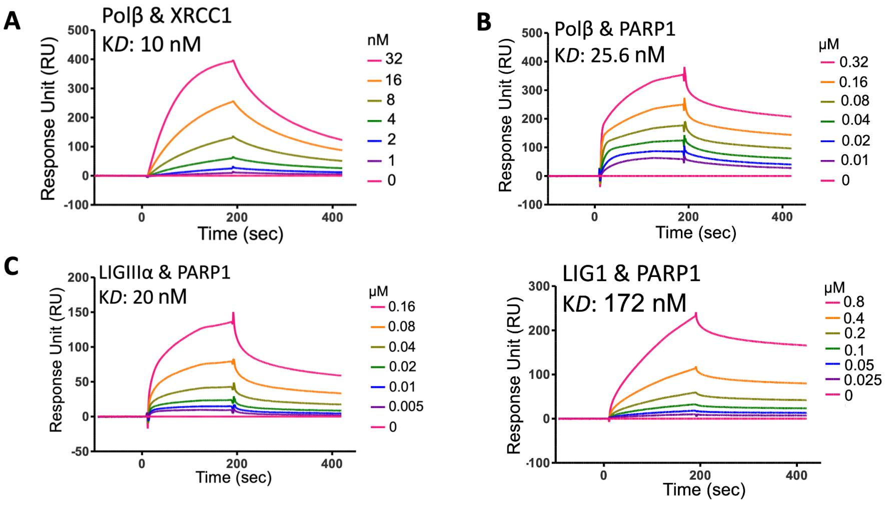

Supplementary Figure 3. **Interaction profile of BER proteins.** Protein-protein interaction measurements in real-time are shown for polβ/XRCC1 (**A**), polβ/PARP1 (**B**), LIGIIIα/PARP1 (**C**), and LIG1/PARP1 (**D**).

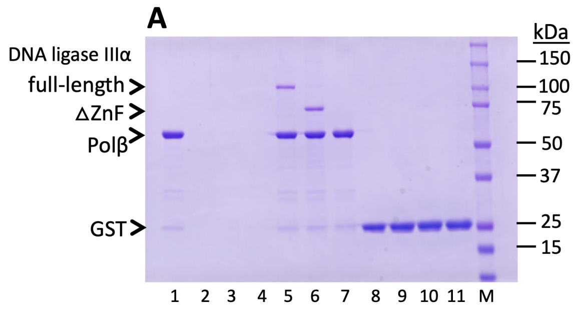

1. Polβ
2. LIGIIIα full-length
3. LIGIIIαΔZnF
4. LIGIIIαΔBRCT
5. Polβ/LIGIIIα full-length
6. Polβ/LIGIIIαΔZnF
7. Polβ/LIGIIIαΔBRCT
8. GST
9. GST/LIGIIIα full-length
10. GST/LIGIIIαΔZnF
11. GST/LIGIIIαΔBRCT

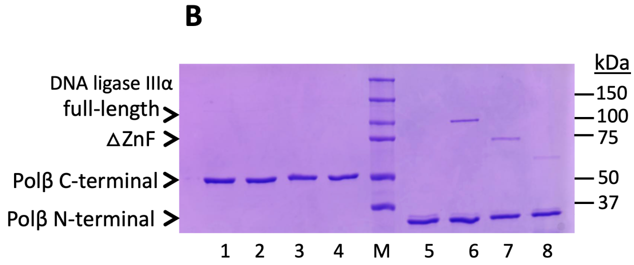

1. Polβ C-terminal
2. Polβ C-terminal/LIGIIIα full-length
3. Polβ C-terminal/LIGIIIαΔZnF
4. Polβ C-terminal/LIGIIIαΔBRCT
5. Polβ N-terminal
6. Polβ N-terminal/LIGIIIα full-length
7. Polβ N-terminal/LIGIIIαΔZnF
8. Polβ N-terminal/LIGIIIαΔBRCT

Supplementary Figure 4. **GST-pull down analyses of polβ and LIGIIIα interaction. (A)** GST-pull down analyses for the interaction of GST-tag polβ full-length protein with his-tag LIGIIIα full-length, truncated mutants LIGIIIαΔZnF and LIGIIIαΔBRCT proteins. The result demonstrates a complex formation of polβ with LIGIIIα full-length and LIGIIIαΔZnF (lanes 5 and 6). There was no binding of polβ full-length with LIGIIIαΔBRCT (line 7). In the control reaction, no binding of GST-tag alone with LIGIIIα proteins tested was confirmed (lanes 9-11). **(B)** GST-pull down analyses for the interaction of GST-tag polβ N-terminal and C-terminal proteins with his-tag LIGIIIα full-length, truncated mutants LIGIIIαΔZnF and LIGIIIαΔBRCT proteins. The result demonstrates a complex formation of polβ N-terminal with LIGIIIα full-length and LIGIIIαΔZnF (lanes 6 and 7). There was no binding of polβ N-terminal with LIGIIIαΔBRCT (line 8). No protein complex formation of polβ C-terminal with LIGIIIα proteins tested was observed (lanes 2-4). M represents a Precision Plus Protein Dual Color Standard (10-250 kDa).

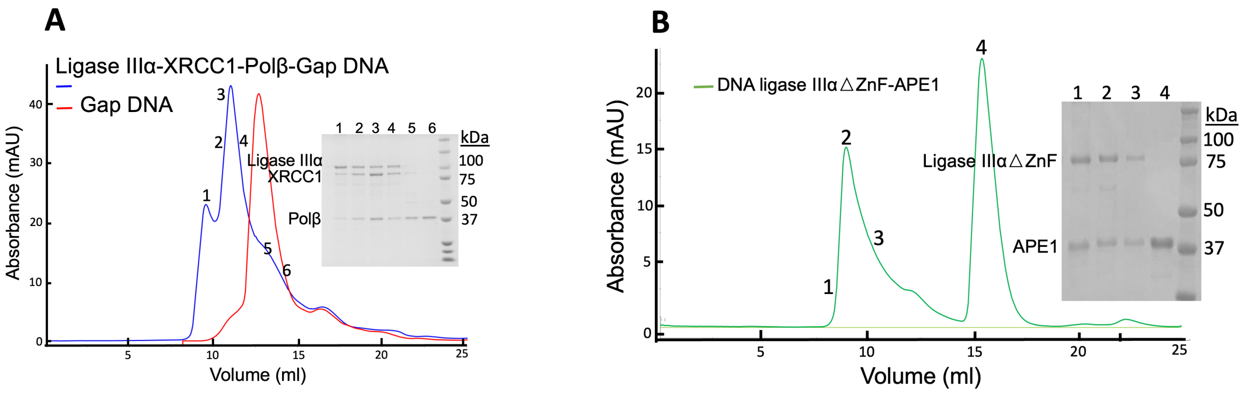

Supplementary Figure 5. BER multi-protein complex formations of LIGIIIα. The protein complex formations were performed through the size exclusion chromatography (SEC) and the elution peaks for individual proteins and the BER multi-protein complex are shown for the BER multi-protein complex of polβ, XRCC1, and LIGIIIα in the presence of gap DNA (**A**) and APE1 and LIGIIIα truncated mutant lacking ZnF domain, LIGIIIαΔZnF (**B**).

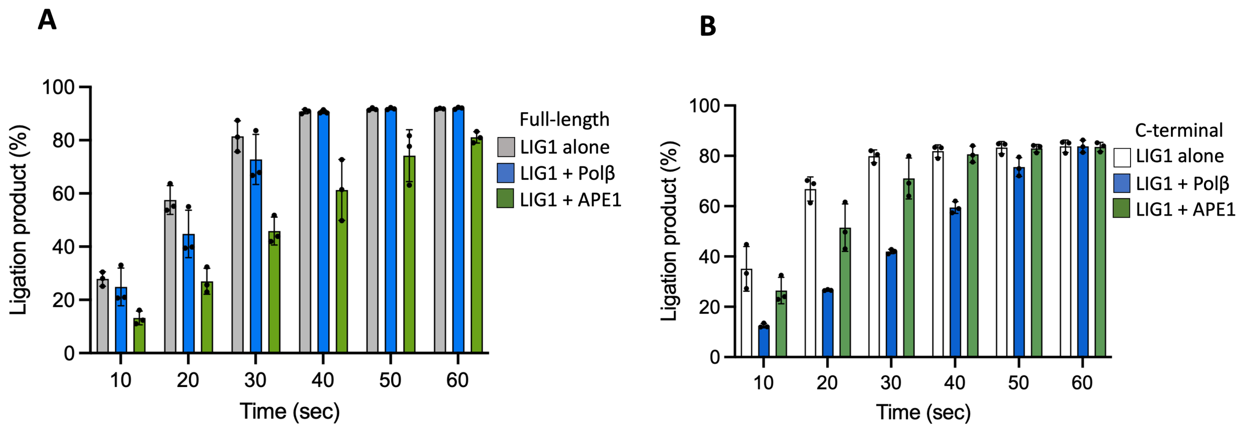

Supplementary Figure 6. Comparison of ligation products by LIG1 in the absence and presence of polβ or APE1. (**A-B**) Graphs show time-dependent changes in the amount of ligation products and the data represent the average of three independent experiments ± SD.

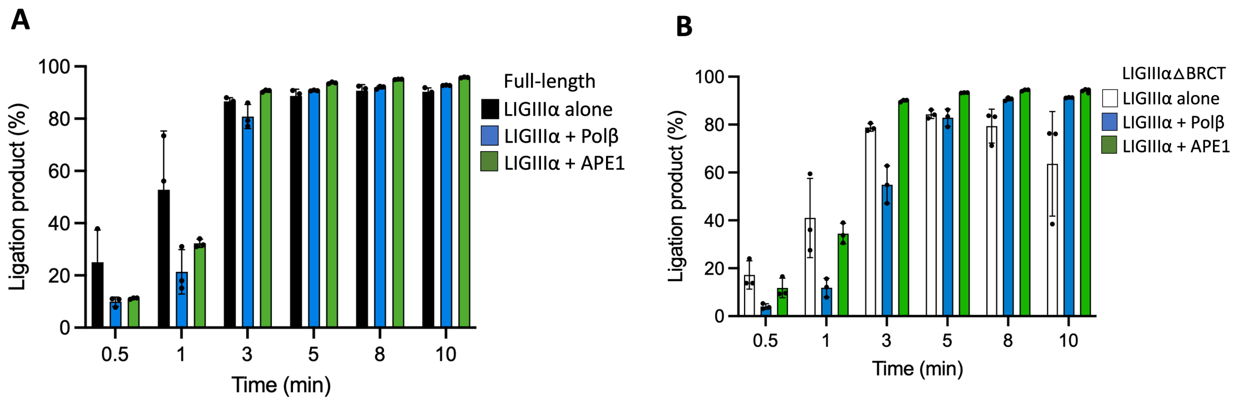

Supplementary Figure 7. **Comparison of ligation products by LIGIIIα in the absence and presence of polβ or APE1.** (**A-B**) Graphs show time-dependent changes in the amount of ligation products and the data represent the average of three independent experiments ± SD.

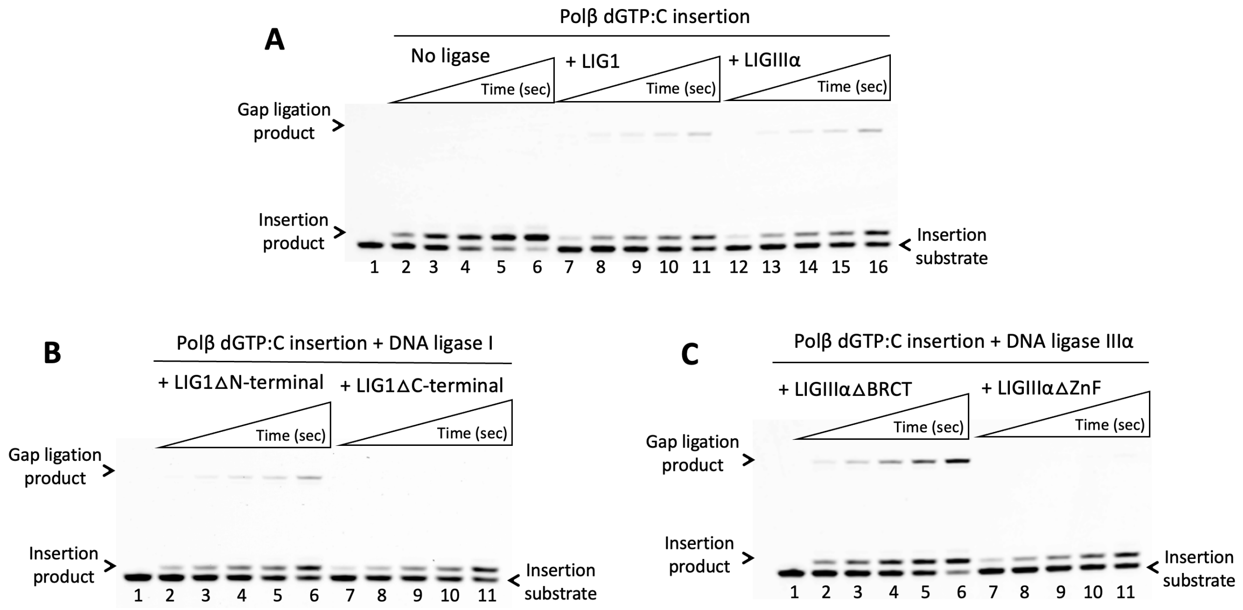

Supplementary Figure 8. **Polβ nucleotide insertion in the absence and presence of BER ligases.** (**A**) Line 1 is the negative enzyme control of the one nucleotide gap DNA substrate. Lanes 2-6, 7-11, and 12-16 are polβ dGTP:C insertion products in the absence and the presence of LIG1 and LIGIIIα, respectively. (**B**) Line 1 is the negative enzyme control of the one nucleotide gap DNA substrate. Lanes 2-6 and 7-11 are polβ dGTP:C insertion products in the presence of LIG1 C-terminal region (ΔN-terminal mutant) and N-terminal region (ΔC-terminal mutant), respectively. (**C**) Line 1 is the negative enzyme control of the one nucleotide gap DNA substrate. Lanes 2-6 and 7-11 are polβ dGTP:C insertion products in the presence of LIGIIIα truncated mutants lacking BRCT (LIGIIIαΔBRCT) and ZnF (LIGIIIαΔZnF) domains, respectively.

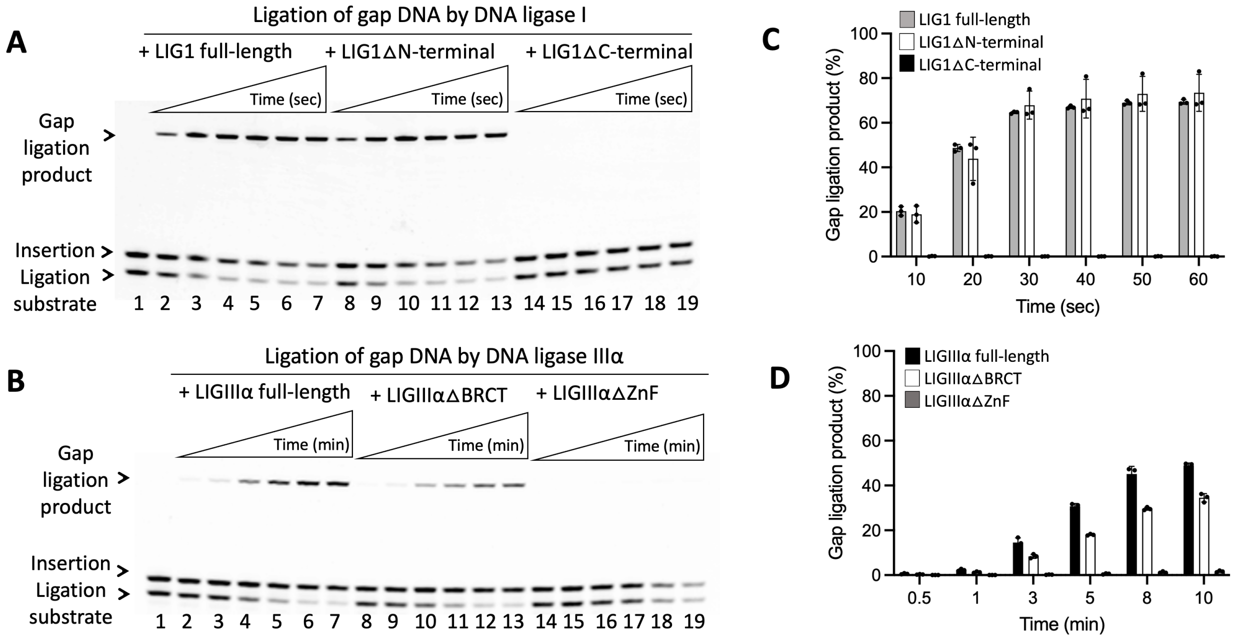

Supplementary Figure 9. **Ligation of gap DNA by BER ligases.** (**A**) Line 1 is the negative enzyme control of the one nucleotide gap DNA substrate. Lanes 2-7, 8-13, 14-19 are the ligation products in the presence of LIG1 full-length and truncated mutants containing C-terminal region (△N-terminal mutant) and N-terminal region (△C-terminal mutant), respectively. (**B**) Line 1 is the negative enzyme control of the one nucleotide gap DNA substrate. Lanes 2-7, 8-13, 14-19 are the ligation products in the presence of LIGIIIα full-length and truncated mutants lacking BRCT (LIGIIIα△BRCT) and ZnF (LIGIIIα△ZnF) domains, respectively. (**C-D**) Graphs show time-dependent changes in the amount of ligation products and the data represent the average of three independent experiments ± SD.

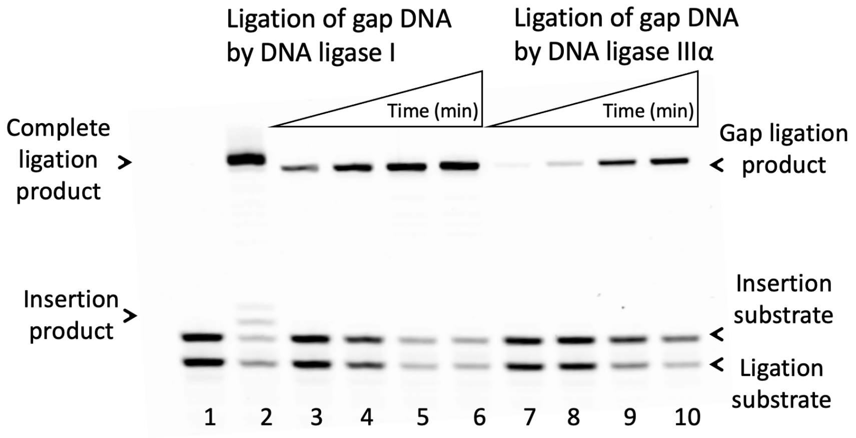

Supplementary Figure 10. **Comparison of complete *versus* gap ligation by BER ligases.** Line 1 is the negative enzyme control of the one nucleotide gap DNA substrate. Line 2 is the positive control showing the complete ligation of nick product after polβ dGTP:C insertion in the coupled reaction. Lanes 3-6 and 7-10 are the gap ligation products of one nucleotide gap DNA by LIG1 and LIGIIIα, respectively.

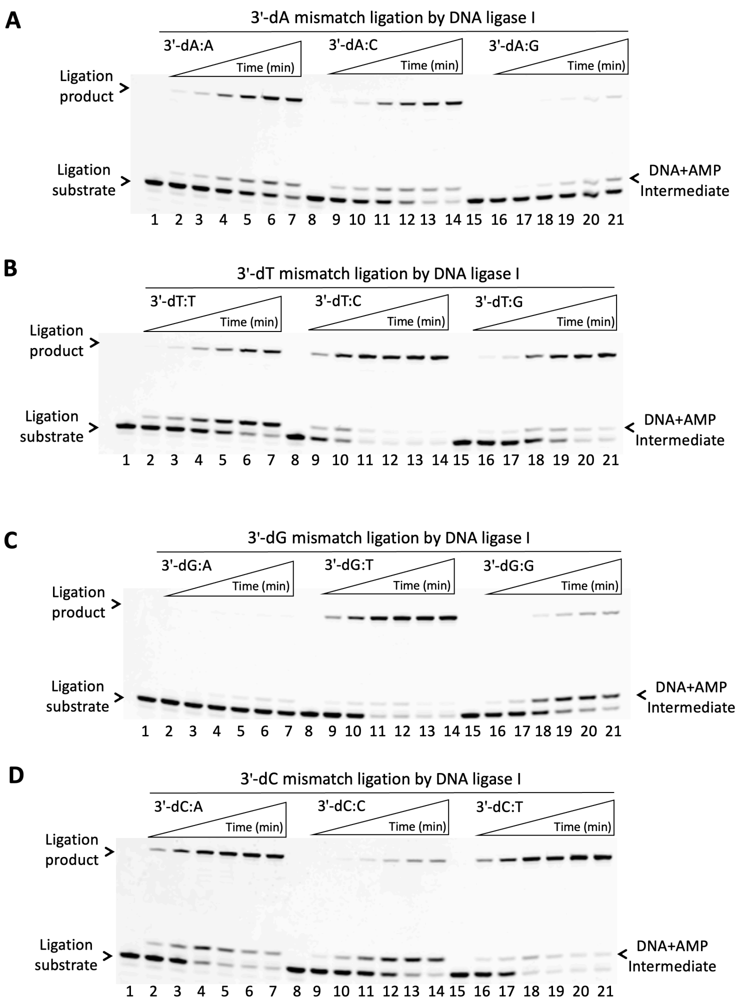

Supplementary Figure 11. **Ligation of nick DNA substrates with all 12 possible mismatches by LIG1.** (**A**) Lanes 1, 8, and 15 are the negative enzyme controls of the nick DNA substrates with corresponding mismatches, respectively. Lanes 2-7, 9-14, 16-21 are the ligation products by LIG1 in the presence of 3'-dA:A, 3'-dA:C, and 3'-dA:G mismatches, respectively, and correspond to time points 0.5, 1, 3, 5, 8, and 10 min. (**B**) Lanes 1, 8, and 15 are the negative enzyme controls of the nick DNA substrates with corresponding mismatches, respectively. Lanes 2-7, 9-14, 16-21 are the ligation products by LIG1 in the presence of 3'-dT:T, 3'-dT:C, and 3'-dT:G mismatches, respectively, and correspond to time points 0.5, 1, 3, 5, 8, and 10 min. (**C**) Lanes 1, 8, and 15 are the negative enzyme controls of the nick DNA substrates with corresponding mismatches,

respectively. Lanes 2-7, 9-14, 16-21 are the ligation products by LIG1 in the presence of 3'-dG:A, 3'-dG:T, and 3'-dG:G mismatches, respectively, and correspond to time points 0.5, 1, 3, 5, 8, and 10 min. (**D**) Lanes 1, 8, and 15 are the negative enzyme controls of the nick DNA substrates with corresponding mismatches, respectively. Lanes 2-7, 9-14, 16-21 are the ligation products by LIG1 in the presence of 3'-dC:A, 3'-dC:C, and 3'-dC:T mismatches, respectively, and correspond to time points 0.5, 1, 3, 5, 8, and 10 min.

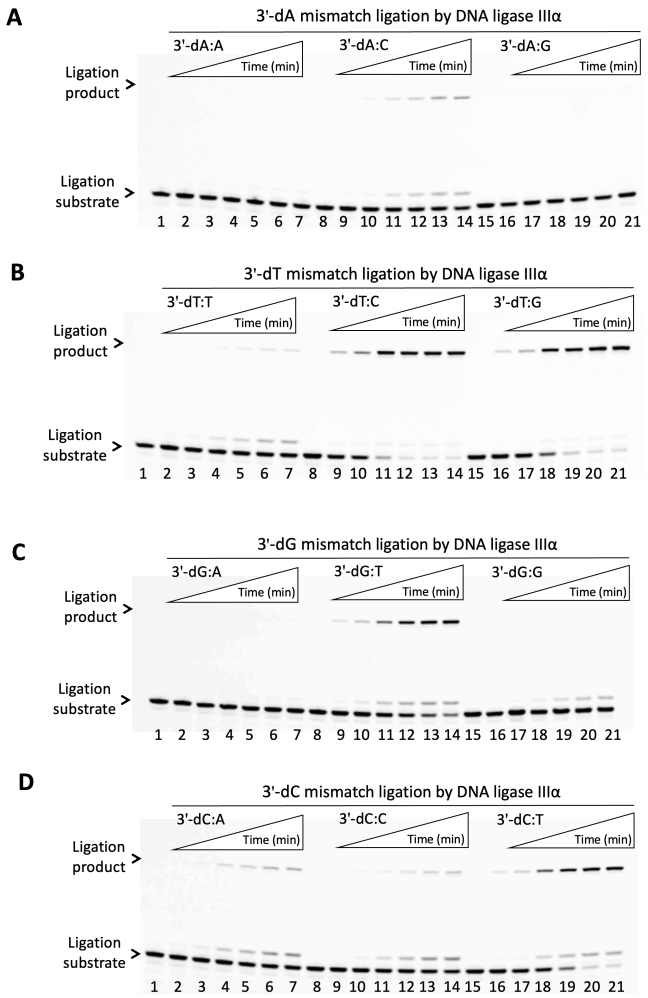

Supplementary Figure 12. **Ligation of nick DNA substrates with all 12 possible mismatches by LIGIIIα.** (**A**) Lanes 1, 8, and 15 are the negative enzyme controls of the nick DNA substrates with corresponding mismatches, respectively. Lanes 2-7, 9-14, 16-21 are the ligation products by LIGIIIα in the presence of 3'-dA:A, 3'-dA:C, and 3'-dA:G mismatches, respectively, and correspond to time points 0.5, 1, 3, 5, 8, and 10 min. (**B**) Lanes 1, 8, and 15 are the negative enzyme controls of the nick DNA substrates with corresponding mismatches, respectively. Lanes 2-7, 9-14, 16-21 are the ligation products by LIGIIIα in the presence of 3'-dT:T, 3'-dT:C, and 3'-dT:G mismatches, respectively, and correspond to time points 0.5, 1, 3, 5, 8, and 10 min. (**C**) Lanes 1, 8, and 15 are the negative enzyme controls of the nick DNA substrates with corresponding

mismatches, respectively. Lanes 2-7, 9-14, 16-21 are the ligation products by LIGIIIα in the presence of 3'-dG:A, 3'-dG:T, and 3'-dG:G mismatches, respectively, and correspond to time points 0.5, 1, 3, 5, 8, and 10 min. (**D**) Lanes 1, 8, and 15 are the negative enzyme controls of the nick DNA substrates with corresponding mismatches, respectively. Lanes 2-7, 9-14, 16-21 are the ligation products by LIGIIIα in the presence of 3'-dC:A, 3'-dC:C, and 3'-dC:T mismatches, respectively, and correspond to time points 0.5, 1, 3, 5, 8, and 10 min.

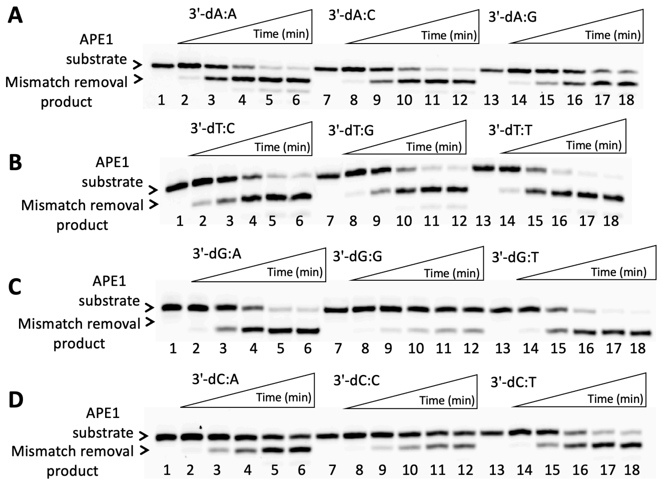

Supplementary Figure 13. **Removal of 3'-mismatched bases from the nick DNA substrates with all 12 possible mismatches by APE1.** (**A**) Lanes 1, 7, and 13 are the negative enzyme controls of the nick DNA substrates with corresponding mismatches, respectively. Lanes 2-6, 8-12, 14-18 are the mismatch removal products by APE1 in the presence of 3'-dA:A, 3'-dA:C, and 3'-dA:G mismatches, respectively, and correspond to time points 1, 3, 5, 8, and 10 min. (**B**) Lanes 1, 7, and 13 are the negative enzyme controls of the nick DNA substrates with corresponding mismatches, respectively. Lanes 2-6, 8-12, 14-18 are the mismatch removal products by APE1 in the presence of 3'-dT:T, 3'-dT:C, and 3'-dT:G mismatches, respectively, and correspond to time points 0.5, 1, 3, 5, 8, and 10 min. (**C**) Lanes 1, 7, and 13 are the negative enzyme controls of the nick DNA substrates with corresponding mismatches, respectively. Lanes 2-6, 8-12, 14-18 are the mismatch removal products by APE1 in the presence of 3'-dG:A, 3'-dG:T, and 3'-dG:G mismatches, respectively, and correspond to time points 0.5, 1, 3, 5, 8, and 10 min. (**D**) Lanes 1, 7, and 13 are the negative enzyme controls of the nick DNA substrates with corresponding mismatches, respectively. Lanes 2-6, 8-12, 14-18 are the mismatch removal products by APE1 in the presence of 3'-dC:A, 3'-dC:C, and 3'-dC:T mismatches, respectively, and correspond to time points 0.5, 1, 3, 5, 8, and 10 min.

DNA Substrates	Sequence
Gap DNA	5'-CATGGGCGGCATGAACC GAGGCCCATCCTCACC-3'-Biotin<br>3'-GTACCCGCCGTACTTGGCCTCCGGGTAGGAGTGG-5'
Nick DNA	5'-CATGGGCGGCATGAACCGGAGGCCCATCCTCACC-3'-Biotin<br>3'-GTACCCGCCGTACTTGGCCTCCGGGTAGGAGTGG-5'

Supplementary Table 1. Gap and nick DNA substrates used in the DNA binding measurements. Nick DNA substrate with preinserted 3'-dG:C and 3'-Biotin as well as one nucleotide gap DNA substrate with template base C and 3'-Biotin were used in BLI-based DNA binding measurements. The base at the template position is underlined. Both DNA substrates include 3'-OH and 5'-P termini.

DNA Substrates	Sequence
Gap DNA (nucleotide insertion)	FAM-5'-CATGGGCGGCATGAACC GAGGCCCATCCTCACC-3'<br>3'-GTACCCGCCGTACTTGGCCTCCGGGTAGGAGTGG-5'
Gap DNA (coupled)	FAM-5'-CATGGGCGGCATGAACC GAGGCCCATCCTCACC-3'-FAM<br>3'-GTACCCGCCGTACTTGGCCTCCGGGTAGGAGTGG-5'

Supplementary Table 2. Gap DNA substrates used in the study. One nucleotide gap DNA substrates with template base C and 5'-FAM label was used in the polβ dGTP:C nucleotide insertion assays. One nucleotide gap DNA substrate with template base C and FAM labels at both 3'- and 5'-ends was used in the coupled assays to test the ligation of the polβ dGTP:C nucleotide insertion products by LIG1 and LIGIIIα. FAM denotes a fluorescent tag and the base at the template position is underlined.

Nick DNA Substrates	Sequence
3'-dA:A	5'-CATGGGCGGCATGAACC**A**GAGGCCCATCCTCACC-FAM-3'<br>3'-GTACCCGCCGTACTTGGACTCCGGGTAGGAGTGG-5'
3'-dG:A	5'-CATGGGCGGCATGAACC**G**GAGGCCCATCCTCACC-FAM-3'<br>3'-GTACCCGCCGTACTTGGACTCCGGGTAGGAGTGG-5'
3'-dC:A	5'-CATGGGCGGCATGAACC**C**GAGGCCCATCCTCACC-FAM-3'<br>3'-GTACCCGCCGTACTTGGACTCCGGGTAGGAGTGG-5'
3'-dT:T	5'-CATGGGCGGCATGAACC**T**GAGGCCCATCCTCACC-FAM-3'<br>3'-GTACCCGCCGTACTTGGTCTCCGGGTAGGAGTGG-5'
3'-dG:T	5'-CATGGGCGGCATGAACC**G**GAGGCCCATCCTCACC-FAM-3'<br>3'-GTACCCGCCGTACTTGGTCTCCGGGTAGGAGTGG-5'
3'-dC:T	5'-CATGGGCGGCATGAACC**C**GAGGCCCATCCTCACC-FAM-3'<br>3'-GTACCCGCCGTACTTGGTCTCCGGGTAGGAGTGG-5'
3'-dA:G	5'-CATGGGCGGCATGAACC**A**GAGGCCCATCCTCACC-FAM-3'<br>3'-GTACCCGCCGTACTTGGGCTCCGGGTAGGAGTGG-5'
3'-dT:G	5'-CATGGGCGGCATGAACC**T**GAGGCCCATCCTCACC-FAM-3'<br>3'-GTACCCGCCGTACTTGGGCTCCGGGTAGGAGTGG-5'
3'-dG:G	5'-CATGGGCGGCATGAACC**G**GAGGCCCATCCTCACC-FAM-3'<br>3'-GTACCCGCCGTACTTGGGCTCCGGGTAGGAGTGG-5'
3'-dA:C	5'-CATGGGCGGCATGAACC**A**GAGGCCCATCCTCACC-FAM-3'<br>3'-GTACCCGCCGTACTTGGCCTCCGGGTAGGAGTGG-5'
3'-dT:C	5'-CATGGGCGGCATGAACC**T**GAGGCCCATCCTCACC-FAM-3'<br>3'-GTACCCGCCGTACTTGGCCTCCGGGTAGGAGTGG-5'
3'-dC:C	5'-CATGGGCGGCATGAACC**C**GAGGCCCATCCTCACC-FAM-3'<br>3'-GTACCCGCCGTACTTGGCCTCCGGGTAGGAGTGG-5'
3'-dG:C	5'-CATGGGCGGCATGAACC**G**GAGGCCCATCCTCACC-FAM-3'<br>3'-GTACCCGCCGTACTTGGCCTCCGGGTAGGAGTGG-5'

Supplementary Table 3. Nick DNA substrates used in ligation assays. Nick DNA substrates with preinserted 3'-dG:C and all 12 possible mismatches were used in the ligation assays. FAM denotes a fluorescent tag and is located at 3'-end of DNA substrates. The base at the template position is underlined and the 3'-deoxyribonucleotide is shown in bold.

Nick DNA Substrates	Sequence
3'-dA:A	5'-FAM-CATGGGCGGCATGAACC**A**GAGGCCCATCCTCACC-3'<br>3'-GTACCCGCCGTACTTGGACTCCGGGTAGGAGTGG-5'
3'-dG:A	5'-FAM-CATGGGCGGCATGAACC**G**GAGGCCCATCCTCACC-3'<br>3'-GTACCCGCCGTACTTGGACTCCGGGTAGGAGTGG-5'
3'-dC:A	5'-FAM-CATGGGCGGCATGAACC**C**GAGGCCCATCCTCACC-3'<br>3'-GTACCCGCCGTACTTGGACTCCGGGTAGGAGTGG-5'
3'-dT:T	5'-FAM-CATGGGCGGCATGAACC**T**GAGGCCCATCCTCACC-3'<br>3'-GTACCCGCCGTACTTGGTCTCCGGGTAGGAGTGG-5'
3'-dG:T	5'-FAM-CATGGGCGGCATGAACC**G**GAGGCCCATCCTCACC-3'<br>3'-GTACCCGCCGTACTTGGTCTCCGGGTAGGAGTGG-5'
3'-dC:T	5'-FAM-CATGGGCGGCATGAACC**C**GAGGCCCATCCTCACC-3'<br>3'-GTACCCGCCGTACTTGGTCTCCGGGTAGGAGTGG-5'
3'-dA:G	5'-FAM-CATGGGCGGCATGAACC**A**GAGGCCCATCCTCACC-3'<br>3'-GTACCCGCCGTACTTGGGCTCCGGGTAGGAGTGG-5'
3'-dT:G	5'-FAM-CATGGGCGGCATGAACC**T**GAGGCCCATCCTCACC-3'<br>3'-GTACCCGCCGTACTTGGGCTCCGGGTAGGAGTGG-5'
3'-dG:G	5'-FAM-CATGGGCGGCATGAACC**G**GAGGCCCATCCTCACC-3'<br>3'-GTACCCGCCGTACTTGGGCTCCGGGTAGGAGTGG-5'
3'-dA:C	5'-FAM-CATGGGCGGCATGAACC**A**GAGGCCCATCCTCACC-3'<br>3'-GTACCCGCCGTACTTGGCCTCCGGGTAGGAGTGG-5'
3'-dT:C	5'-FAM-CATGGGCGGCATGAACC**T**GAGGCCCATCCTCACC-3'<br>3'-GTACCCGCCGTACTTGGCCTCCGGGTAGGAGTGG-5'
3'-dC:C	5'-FAM-CATGGGCGGCATGAACC**C**GAGGCCCATCCTCACC-3'<br>3'-GTACCCGCCGTACTTGGCCTCCGGGTAGGAGTGG-5'

Supplementary Table 4. Nick DNA substrates used in APE1 assays. Nick DNA substrates with preinserted all 12 possible mismatches were used in APE1 exonuclease assays. FAM denotes a fluorescent tag and is located at 5'-end of DNA substrates. The base at the template position is underlined and the 3'-deoxyribonucleotide is shown in bold.

Nick DNA Substrates	Sequence
3'-dA:A	5'-FAM-CATGGGCGGCATGAACC**A**GAGGCCCATCCTCACC-3'-FAM<br>3'-GTACCCGCCGTACTTGGACTCCGGGTAGGAGTGG-5'
3'-dG:A	5'-FAM-CATGGGCGGCATGAACC**G**GAGGCCCATCCTCACC-3'-FAM<br>3'-GTACCCGCCGTACTTGGACTCCGGGTAGGAGTGG-5'
3'-dC:A	5'-FAM-CATGGGCGGCATGAACC**C**GAGGCCCATCCTCACC-3'-FAM<br>3'-GTACCCGCCGTACTTGGACTCCGGGTAGGAGTGG-5'
3'-dT:T	5'-FAM-CATGGGCGGCATGAACC**T**GAGGCCCATCCTCACC-3'-FAM<br>3'-GTACCCGCCGTACTTGGTCTCCGGGTAGGAGTGG-5'
3'-dG:T	5'-FAM-CATGGGCGGCATGAACC**G**GAGGCCCATCCTCACC-3'-FAM<br>3'-GTACCCGCCGTACTTGGTCTCCGGGTAGGAGTGG-5'
3'-dC:T	5'-FAM-CATGGGCGGCATGAACC**C**GAGGCCCATCCTCACC-3'-FAM<br>3'-GTACCCGCCGTACTTGGTCTCCGGGTAGGAGTGG-5'
3'-dA:G	5'-FAM-CATGGGCGGCATGAACC**A**GAGGCCCATCCTCACC-3'-FAM<br>3'-GTACCCGCCGTACTTGGGCTCCGGGTAGGAGTGG-5'
3'-dT:G	5'-FAM-CATGGGCGGCATGAACC**T**GAGGCCCATCCTCACC-3'-FAM<br>3'-GTACCCGCCGTACTTGGGCTCCGGGTAGGAGTGG-5'
3'-dG:G	5'-FAM-CATGGGCGGCATGAACC**G**GAGGCCCATCCTCACC-3'-FAM<br>3'-GTACCCGCCGTACTTGGGCTCCGGGTAGGAGTGG-5'
3'-dA:C	5'-FAM-CATGGGCGGCATGAACC**A**GAGGCCCATCCTCACC-3'-FAM<br>3'-GTACCCGCCGTACTTGGCCTCCGGGTAGGAGTGG-5'
3'-dT:C	5'-FAM-CATGGGCGGCATGAACC**T**GAGGCCCATCCTCACC-3'-FAM<br>3'-GTACCCGCCGTACTTGGCCTCCGGGTAGGAGTGG-5'
3'-dC:C	5'-FAM-CATGGGCGGCATGAACC**C**GAGGCCCATCCTCACC-3'-FAM<br>3'-GTACCCGCCGTACTTGGCCTCCGGGTAGGAGTGG-5'

Supplementary Table 5. Nick DNA substrates used in APE1/DNA ligase coupled assays. Nick DNA substrates with preinserted all 12 possible mismatches were used in the coupled assays to test the ligation of APE1 removal products by LIG1 or LIGIIIα. FAM denotes a fluorescent tag and is located at 5'-end of DNA substrates. The base at the template position is underlined and the 3'-deoxyribonucleotide is shown in bold.

**Protein interactions of BER ligases**	**K*D* (nM)**
Polβ/LIG1 full-length	142 ± 13.5
Polβ/LIG1△N-terminal	127 ± 25
Polβ/LIG1△C-terminal	ND
Polβ/LIGIIIα full-length	7.0 ± 1.0
Polβ/LIGIIIα△ZnF	27.0 ± 5.0
Polβ/LIGIIIα△BRCT	520 ± 58
APE1/LIG1 full-length	117 ± 2.3
APE1/LIG1△N-terminal	28 ± 1.5
APE1/LIG1△C-terminal	ND
APE1/LIGIIIα full-length	3.3 ± 1.1
APE1/LIGIIIα△ZnF	4.9 ± 1.2
APE1/LIGIIIα△BRCT	1048 ± 10.2

Supplementary Table 6. Protein-protein interaction profile of BER ligases. Protein interactions of BER ligases, LIG1 and LIGIIIα, is shown for polβ and APE1. The table shows the interaction regions for LIG1 full-length and truncated mutants containing N-terminal (LIG1△C-terminal) and C-terminal (LIG1△N-terminal) as well as LIGIIIα full-length and truncated mutants lacking Zn-finger (LIGIIIα△ZnF) and BRCT (LIGIIIα△BRCT) domains.

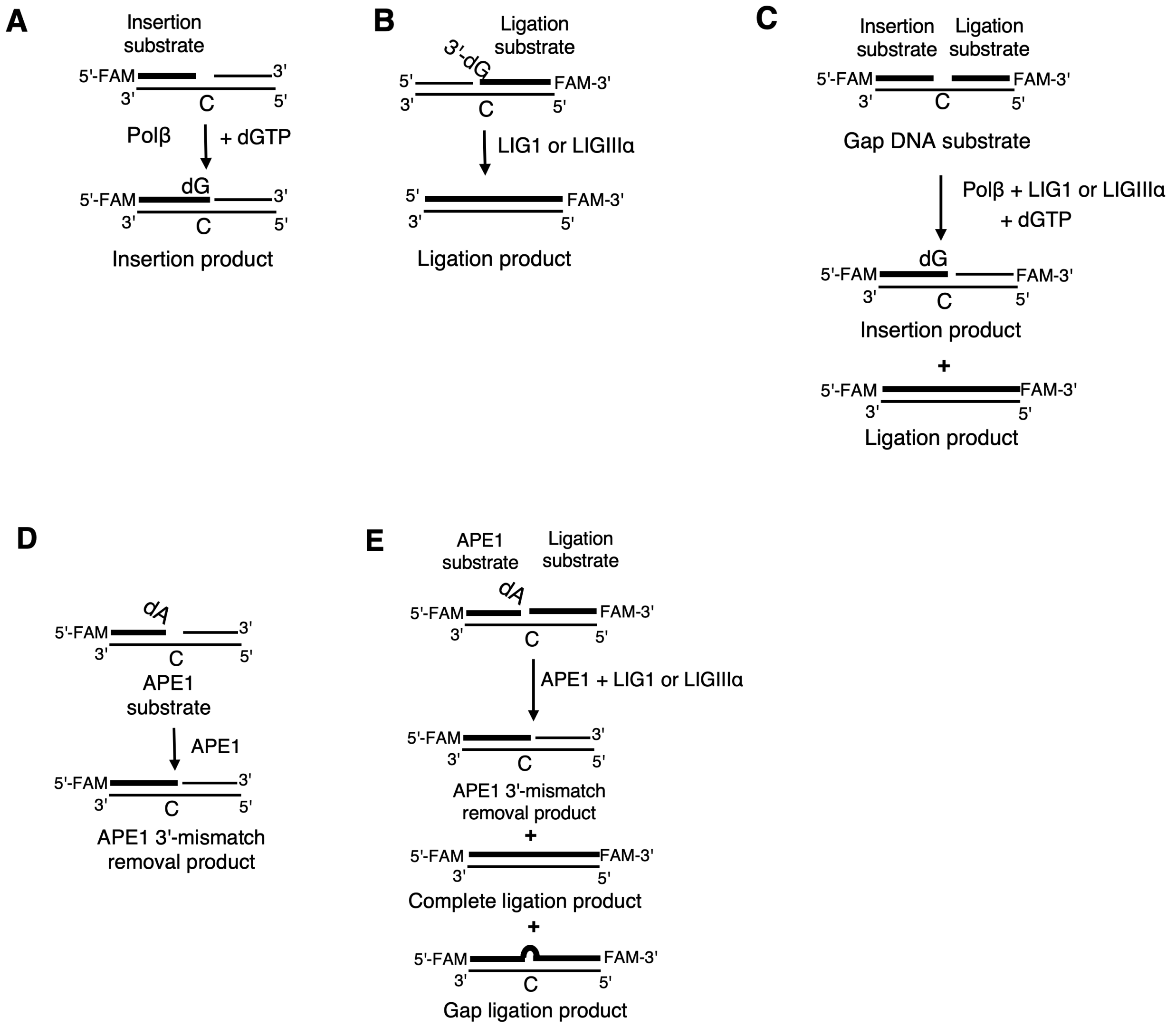

Supplementary Scheme 1. DNA repair assays used in this study. (**A**) The insertion assay was used to test polβ dGTP:C insertion in the absence and presence of DNA ligase. (**B**) The ligation assay was used to test the nick sealing efficiency of LIG1 or LIGIIIα in the absence and presence of polβ or APE1. (**C**) The coupled assay was used to test the ligation of polβ dGTP:C insertion products by LIG1 or LIGIIIα simultaneously in the same reaction mixture. (**D**) The exonuclease removal assay was used to test the efficiency of 3'-mismatch removal by APE1 from nick DNA substrate. (**E**) The coupled assay was used to test the efficiency of 3'-mismatch removal by APE1 and the nick sealing efficiency of LIG1 or LIGIIIα simultaneously in the same reaction mixture.

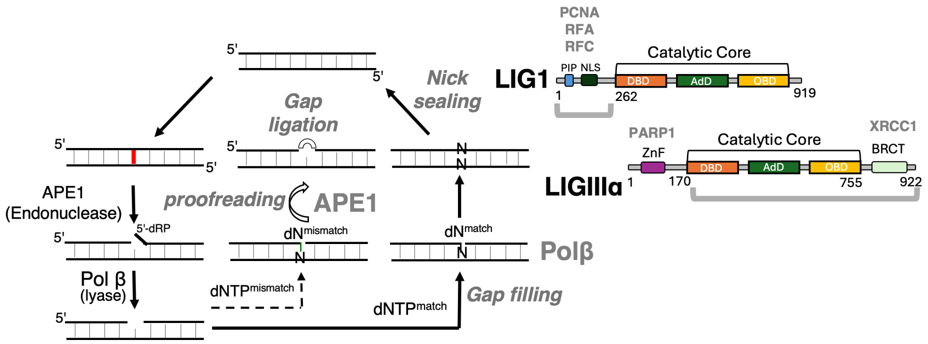

Supplementary Scheme 2. Illustration of the substrate-product channeling mechanism between APE1, polβ, and DNA ligases at the downstream steps of BER pathway. The model shows the coordination between BER ligases (LIG1 and LIGIIIα) with APE1 and polβ at the downstream steps of the repair pathway. Scheme shows the regions of BER ligases that mediate protein interactions with APE1 and polβ as shown in grey brackets; N-terminal non-catalytic domain of LIG1 and the region corresponding to the catalytic core and BRCT domain of LIGIIIα. BER ligases can seal resulting nick repair product in the presence of a correct nucleotide incorporated by polβ during gap filling step of the repair pathway. In case of polβ mismatch incorporation, the product of nick repair intermediate containing non-canonical ends can serve as a substrate for APE1 proofreading function leading to the removal of 3'-mismatched base and subsequent ligation of resulting gap repair intermediate by BER ligases. LIG1 and LIGIIIα contain the N-terminal DNA binding domain (DBD) and the C-terminal catalytic core consisting of the adenylation domain (AdD) and the oligonucleotide binding domain (OBD). LIGIIIα includes an N-terminal zinc finger (ZnF) and a breast and ovarian cancer susceptibility protein-1 C-terminal domain (BRCT). LIG1 has a nuclear localization signal (NLS), the N-terminal region containing a replication factory targeting sequence also known as a PCNA-interacting peptide (PIP) box.
